# The effect of different combinations of open invitations and timed appointments on breast screening attendance: service evaluation of invitation strategies in the NHS Breast Screening Programme

**DOI:** 10.1038/s41416-026-03436-8

**Published:** 2026-05-14

**Authors:** Shuping J. Li, Adam R. Brentnall, Jacqui Cookson, Sue Hudson, Samantha L. Quaife, Sharon Webb, Emma O’Sullivan, Jacquie Jenkins, Jo Waller, Stephen W. Duffy, Judith Offman

**Affiliations:** 1 Centre for Cancer Screening, Prevention and Early Diagnosis, Wolfson Institute of Population Health, Queen Mary University of London, London, UK; 2 NHS Breast Screening Programme, Primary Care, Community, Vaccinations & Screening (PCVS) Directorate, NHS England, London, UK; 3 Peel & Schriek Consulting Ltd, London, UK

**Keywords:** Health policy, Population screening, Epidemiology

## Abstract

**Background:**

The NHS Breast Screening Programme invites eligible women using either a timed appointment letter (option to change), or an open invitation to make an appointment. Non-attenders receive a ‘Timed’ or ‘Open’ second invitation. NHS England commissioned a service evaluation to determine the effect of different combinations of timed and open invitations.

**Methods:**

Seven services, selected to ensure adequate representation of diverse socio-economic, ethnic, urban/rural, and current uptake groups, participated. Women were individually quasi-randomised to four combinations of Open/Timed invitations. The primary outcome was 90-day attendance.

**Results:**

17,965 women (mean age 58 years, IQR: 47–69) invited from April-October 2023 were included. Attendance overall increased from 49.1% (95%CI 47.7–50.6%) for Open/Open to 67.9% (66.5–69.2%) for Timed/Timed. Attendance following Open/Timed or Timed/Open invitations was 60.0% (58.6–61.4%) and 64.7% (63.3–66.1%) respectively. The same pattern was observed across all deprivation quintiles. Attendance amongst the most deprived increased from 41.1% (38.2–44.1%; Open/Open) to 63.3% (60.6–66.2%; Timed/Timed), compared with 61.4% (57.6–65.2%) to 78.0% (74.5–81.4%) for the least deprived quintile.

**Conclusion:**

Sending timed appointment invitations increases attendance at breast screening. This might have a larger impact in the most deprived areas. Findings informed NHS England on the most effective invitation methodologies from April 2025.

## Background

In England, the NHS Breast Screening Programme (NHSBSP) invites people registered with their GP as female aged 50–53 up to 71 years for breast screening every 3 years [1]. In addition to this routine screening pathway, very high risk women are offered more regular screening, usually on an annual basis. An effective screening programme requires high uptake [2]. In England, uptake is defined as attendance within 6 months of first invitation, and the acceptable level is 70%+. Since the Covid-19 pandemic, breast screening uptake in England has fallen below this standard and has yet to fully recover to pre-Covid pandemic levels (62.4% for 2021/22, 64.6% for 2022/23, and 70.0% for 2023/24) [1, 3, 4].

Prior to the Covid-19 pandemic, every woman received an invitation letter with a specified date, time and place for screening (referred to as ‘timed appointment’ by the NHSBSP). The usual practice for second (reminder) invitations for non-attenders varied by screening services. Some services also sent second timed appointment letters, whereas others sent invitation letters asking women to contact the service to make an appointment (open invite). To help reduce the post-Covid screening backlog and make best use of screening appointments, a national decision was made for all services to switch to open invitations for both first and second invitation letters [5]. Subsequently, some services have returned to using timed appointments. At the time of the service evaluation reported in this paper, various combinations of timed appointments and open invites were used by different services.

The European Commission Initiative of Breast Cancer recommend using a letter with a fixed appointment to invite women to screening, but they do not specify whether reminder letters should also be with a fixed appointment [6]. Several studies have looked at how different invitation strategies may affect breast screening participation, with timed appointments associated with a higher uptake [7, 8]. However, there is limited information on how different combinations of first and/or second invitations affect attendance, particularly across the diverse population served by the NHSBSP. Even though all four combinations of invitations are currently used by different screening services, it is difficult to robustly evaluate the different strategies retrospectively due to confounding factors including differences between screening catchment areas with regards to ethnicity, deprivation, pre-existing uptake level, and rural vs. urban settings. A prospective service evaluation was therefore commissioned by NHS England (NHSE) to reliably determine the effect of different combinations of timed appointments and open invitations on attendance rate to inform future service delivery.

## Methods

### Evaluation design and participants

Seven NHSBSP services took part in this evaluation. They were selected to ensure adequate representation of diverse socio-economic, ethnic, urban/rural, and current uptake groups (Supplementary Table 1). The services were: Central and East London, North London, Outer Northeast London, Dudley, Wolverhampton & Southwest Staffordshire, Cambridge, Doncaster, and North and East Devon.

Women are invited to breast screening in batches of varying sizes, ranging from several hundred to over one thousand. Batches were eligible for this evaluation if breast screening dates fell within the study period (April to October 2023). Individual women within a batch were excluded from the evaluation if they had opted out permanently from the screening programme, had self-referred for screening, required special appointments (e.g., due to learning disability), were on an early recall protocol, or were part of high-risk screening.

### Invitation strategy assignment

Women were quasi-randomised to four invitation strategies based on a unique identifier used by the NHSBSP (SX number), and their NHS number. The first invitation letter used an open invitation or a timed appointment based on the last digit of their SX number being odd/even. If required, second invitation letters were open or timed appointments based on the tenth digit of the NHS number being odd/even. There were four invitation strategies: (1) Timed for both the first and second invitation (Timed/Timed), (2) Open invitations for both (Open/Open), (3) Timed followed by an Open invitation (Timed/Open), and (4) Open followed by a Timed invitation (Open/Timed). Each service was assigned a different way to interpret odd/even of each digit for open/timed, to balance overall allocation across services (Graeco-Latin squares).

Screening centre staff could not be blinded as they were processing invitations. However, researchers overseeing and conducting the statistical analysis remained blinded until the statistical analysis plan was completed and the final dataset extracted. As this was an evaluation of standard practices participants were not aware of their inclusion.

### Procedures

All participating screening services had experience using timed appointments and open invitation letters. Standard NHSBSP letter templates were used for first invitations. Timed invitation letters included a telephone number to call if the appointment was not convenient, as is standard practice. Women with an open invitation letter were asked to book their appointment by phone or, where available, online. All participating screening services sent text message reminders following local standard practice: mobile phone numbers are obtained either at previous appointments or, for non-attenders or prevalent women, numbers are downloaded from primary care. Most centres sent reminders about 48 h before a screening appointment. All women being invited for their first (prevalent) screen received a letter tailored for this and an information leaflet. Some but not all women who were invited for a subsequent screening visit (incident) received an information leaflet. Monitoring was carried out by NHSE to ensure women were booked into the correct clinics and received the correct invitation letter.

The criteria to identify those to be sent a second invitation were: (i) if timed appointment letter: did not attend on the day of appointment; (ii) if open invitation letter: did not book an appointment within 4 weeks of receiving their first open letter. Women who had opted out of screening in this screening round did not receive a second invitation of any kind, but remained in the study as non-attenders. Follow-up for attendance was until the 19th of April 2024.

### Outcomes

The primary endpoint was 90-day attendance. For women with a first timed appointment, this was defined as attending the originally offered screening appointment, or an alternative appointment within 90 days of the original appointment. As we were interested in the impact of the different invitation strategies on attendance, we also included women where the screening test was not completed. For open invitations, the NHSBSP National Breast Screening IT System (NBSS) uses the concept of a “holding clinic” to pseudo-schedule appointments for the purpose of entering women onto the NBSS system and sending them their invitation letter. Once a woman calls to book an appointment, they are moved out of the holding clinic into a real clinic. If a woman does not book an appointment or informs the screening service that she does not want an appointment, they are considered non-attenders after their ‘holding clinic appointment’. Women who did not book will receive a second invitation. The original appointment was defined as the date of the holding clinic, which is about four weeks after the date of the invitation. Two-time non-attenders do not receive any further invitations until the next screening round, when they will be sent the standard first invitation letter.

The secondary endpoint was 180-day attendance, defined as attending the originally offered screening appointment, or an alternative appointment within 180 days of the original. For open invitations, the originally offered date was the date of the initial holding clinic. An exploratory outcome used time since letter printed and sent until last attendance or last follow-up (Supplementary methods).

### Statistical analysis

A statistical analysis plan was finalised prior to analysis (Supplementary Materials).

#### Analysis methods

Attendance rates were compared using Poisson regression with robust variance estimates, and a Wald test. Statistical testing was two-sided, with *p* < 0.05 considered to be statistically significant.

Hierarchical testing was pre-specified (Supplement) and used for the primary analysis. A global likelihood-ratio test with three degrees of freedom was first applied. Then pairwise comparisons were tested in a pre-defined order (only proceeded to test the next pair if the current one was significant): (1) Timed/Timed vs Open/Open, (2) Timed/Timed vs Open/Timed, (3) Timed/Open vs Open/Timed, (4) Timed/Timed vs Timed/Open, and (5) Open/Timed vs Open/Open. Effect sizes are presented with 95% Confidence Intervals (CIs) and *p*-value.

Pre-defined subgroups of main focus were index of multiple deprivation (IMD; quintile), and area-level estimated ethnicity (White vs Not White; see Supplementary materials); also screening service and age group/prevalence status. As prevalence status did not indicate whether a woman was a first non-attender or never attender of subsequent invitations, prevalence status and age was combined into three groups: prevalent and <60, incident and <60 and 60+ prior to analysis. Individual level IMD was obtained using the IMD of the Lower Super Output Area of each woman’s residential postcode.

#### Secondary endpoint methods

The secondary endpoint was attendance 180 days since first offered appointment. The relative risk (RR) between groups was estimated by dividing the results at suitable timepoints, with 95%CI calculated. Non-attendance by the end of follow-up period was considered censored. Stata version 17.0 was used for all analysis [9].

#### Sample size

Previous studies suggested an absolute 8% increase in participation with timed appointment invitations (first letter), compared with open invitations [8]. For a comparison of two groups with participation rates of 65% and 73%, 524 persons per group would give 80% power to detect this difference as significant at 5% level with two-sided testing. To allow comparisons of subgroups we increased the planned sample size to 16,000.

### Role of the funding source

This study was commissioned by NHSE. JC, JJ, EO’S and SW were employed by NHSE and contributed to evaluation design and data collection, the statistical analysis plan, and interpretation of findings. The corresponding author had full access to all the data in the evaluation and had final responsibility for the decision to submit for publication.

### Ethical approval

The UK Health Research Authority, which oversees ethical approval for all research involving NHS patients, does not consider this to be research (see https://www.hra-decisiontools.org.uk/research/docs/DefiningResearch&lt;div class="pi"&gt;show [QJ]Table_Oct2022.pdf). This service evaluation measured the impact of first and second invitation approaches currently being used as standard of care within the programme to find out what improvements can be achieved within the NHSBSP. Furthermore, service evaluations may involve randomisation for sampling, but not interventions. Since we did not allocate new interventions but standard of care invitation strategies, pseudo-randomisation was only used for sampling. As this work was designed as a service evaluation and not research, ethical approval was not required. It was therefore also not registered on any clinical trials databases. We completed a Data Protection Impact Assessment, and fully anonymous data were transferred from NHSE to Queen Mary University of London for analysis, under a data transfer agreement.

## Results

**Fig. 1:**
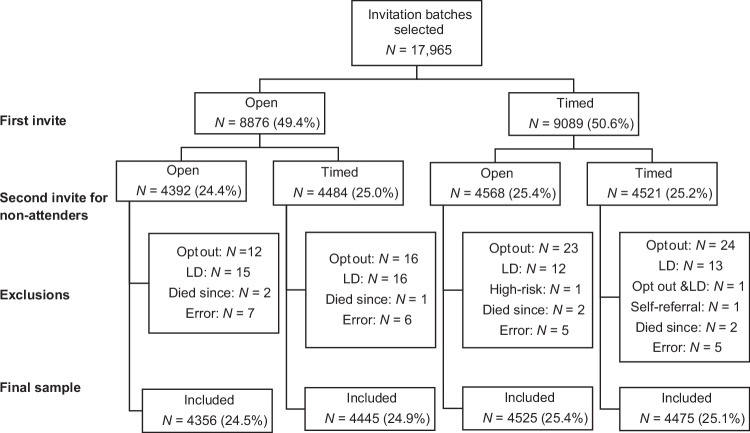
Flow of invitees and participants. Exclusions were applied before the analysis. LD. Learning disability. Opt-out: notified screening service they do not wish to be screened or invited in the future. Error: these women had screening date recorded before their invitation letter was created, or no date for letter creation, print nor screening offered available for obtaining endpoint.

17,965 eligible women were invited including *n* = 8876 open invitations (49%) and 9089 timed appointments (51%) (Fig. 1). Of women allocated open invitations, *n* = 4392 (49%) and 4484 (51%) were assigned to open invitations and timed appointments for their second invitation letter respectively. For the group which received timed appointments, 4568 (50%) and 4521 (50%) women were assigned to open invitations and timed appointments for their second invitation respectively. After excluding 165 (1%) women from evaluation batches for reasons given in methods, *n* = 17,801 women were included for analysis.

Across the four invitation strategy groups, women had similar demographic characteristics (Table 1), with an average age of 58.4 (IQR 11.0) years. Approximately half were from the two most deprived quintiles (24.2% IMD1 and 24.5% IMD2). 24% were below 60 years old and had attended breast screening whereas 32% were below 60 and had not attended screening either due to not having been invited yet or not attending at any previous rounds. 70% of the participants were estimated to be of white ethnicity based on the lower super output area (LSOA) in which they resided. Batches invited by London services were from areas of greater ethnic diversity than those invited by other regions, and had higher levels of socio-economic deprivation (Supplementary Table 2). Allocation to intervention strategies appeared well balanced across all subgroups of interest (Table 1).

**Table 1:** Demographic characteristics of women included in the service evaluation.

	Invitation Group									
	Open/ Open		Open/ Timed		Timed/ Open		Timed/ Timed		Total	
Total	4357		4445		4525		4475		17,802	
Age in years (Mean, Q1–Q3) ^a^	58.3	(58.0–64.0)	58.6	(58.0–64.0)	58.5	(58.0–64.0)	58.4	(58.0–64.0)	58.4	(58.0–64.0)
IMD (quintile)										
1: most deprived	1070	(24.6%)	1049	(23.6%)	1079	(23.8%)	1115	(24.9%)	4313	(24.2%)
2	1041	(23.9%)	1102	(24.8%)	1147	(25.3%)	1077	(24.1%)	4367	(24.5%)
3	776	(17.8%)	876	(19.7%)	809	(17.9%)	828	(18.5%)	3289	(18.5%)
4	813	(18.7%)	839	(18.9%)	850	(18.8%)	861	(19.2%)	3363	(18.9%)
5: least deprived	619	(14.2%)	555	(12.5%)	604	(13.3%)	558	(12.5%)	2336	(13.1%)
missing	38	(0.9%)	24	(0.5%)	36	(0.8%)	36	(0.8%)	134	(0.8%)
Prevalence and age										
prevalent and <60	1397	(32.1%)	1,371	(30.8%)	1440	(31.8%)	1411	(31.5%)	5619	(31.6%)
incident and <60	1058	(24.3%)	1081	(24.3%)	1097	(24.2%)	1104	(24.7%)	4340	(24.4%)
60+	1893	(43.4%)	1979	(44.5%)	1985	(43.9%)	1955	(43.7%)	7812	(43.9%)
missing	9	(0.2%)	14	(0.3%)	3	(0.1%)	5	(0.1%)	31	(0.2%)
Ethnicity^b^										
Asian		10.7%		10.6%		10.6%		10.7%		10.7%
Black		7.6%		7.9%		7.8%		8.1%		7.9%
Mixed		1.4%		1.4%		1.4%		1.4%		1.4%
White		69.7%		69.5%		70.0%		69.3%		69.6%
Other		3.3%		3.4%		3.4%		3.3%		3.4%
Missing^c^		7.3%		7.2%		6.8%		7.2%		7.1%
Services										
Doncaster	673	(15.4%)	664	(14.9%)	687	(15.2%)	686	(15.3%)	2710	(15.2%)
Cambridge	561	(12.9%)	552	(12.4%)	595	(13.1%)	593	(13.3%)	2301	(12.9%)
North London	717	(16.5%)	795	(17.9%)	762	(16.8%)	733	(16.4%)	3007	(16.9%)
Outer NE London	404	(9.3%)	403	(9.1%)	407	(9.0%)	451	(10.1%)	1665	(9.4%)
Central and East London	564	(12.9%)	583	(13.1%)	588	(13.0%)	591	(13.2%)	2326	(13.1%)
North and East Devon	612	(14.0%)	640	(14.4%)	614	(13.6%)	602	(13.5%)	2468	(13.9%)
Dudley,Wolverh and SW Staffords ^d^	826	(19.0%)	808	(18.2%)	872	(19.3%)	819	(18.3%)	3325	(18.7%)

*IMD* index of multiple deprivation, *IQR* interquartile range.

^a^Age in years at first offered appointment.

^b^Ethnicity is estimated based on the LSOA of women’s residential postcode, not of individual woman. See Supplementary for further detail. As they are estimates, only percentages are presented here.

^c^Women whose LSOA was unable to be linked to identify residential region.

^d^Dudley, Wolverhampton, and Southwest Staffordshire.

Use of timed invitations substantially increased breast screening attendance (Table 2). An Open/Open strategy had the lowest attendance and Timed/Timed invitations the highest. In the Open/Open group, approximately half (49.1%, 95% CI 47.7–50.6%) of women attended within 90 days, compared with just over two thirds (67.9%, 95% CI 66.5–69.2%) for Timed/Timed. Timed/Open resulted in higher 90-day attendance (64.7%, 95% CI 63.3–66.1%) than Open/Timed (60.0%, 95% CI 58.6–61.4%). Extending follow-up from 90 to 180 days increased attendance by about 3% in all invitation groups.

**Table 2:** Primary and secondary endpoint by invitation groups.

Evaluation groups	Total invited	90-day attendance	90-day attendance (%)	95%CI	180-day attendance	180-day attendance (%)	95%CI
Open/Open Invitation	4357	2141	49.1%	[47.7%,50.6%]	2321	53.3%	[51.8%,54.8%]
Open/Timed Invitation	4445	2667	60.0%	[58.6%,61.4%]	2822	63.5%	[62.1%,64.9%]
Timed/Open Invitation	4525	2928	64.7%	[63.3%,66.1%]	2989	66.1%	[64.7%,67.4%]
Timed/Timed Invitation	4475	3037	67.9%	[66.5%,69.2%]	3080	68.8%	[67.5%,70.2%]
Total	17,802	10,773	60.5%	[59.8%,61.2%]	2321	63.0%	[62.3%,63.7%]

Table 3 shows the 90-day incidence rate ratio (IRRs) and 180-day attendance relative risks (RR) comparing two invitation strategies at a time. Comparing Timed/Timed to Open/Open, the 90-day attendance was relatively 38% higher (IRR = 1.38, 95% CI 1.33–1.43). When the first invitation was open, having a second timed appointment instead of an open invitation yielded a relative 22% increase (IRR = 1.22, 95%CI 1.17–1.27) in 90-day attendance (Table 2). Having both invitations as a timed appointments instead of an open followed by a timed invitation yielded a relative 13% increase (IRR = 1.13, 95% CI 1.10–1.17) in 90-day attendance. Using Timed/Timed compared to Timed/Open only resulted in a relative 5% increase (IRR = 1.05, 95% CI 1.02–1.08).

**Table 3:** Incidence rate ratio (IRR) at 90-day since first offered appointment by pairwise comparison of two invitation strategies.

	Comparison strategy	vs	Baseline strategy	90-day attendance			180-day attendance	
				IRR	95% CI	*p*-value	RR	95% CI
1	Timed/ Timed	vs	Open/ Open	1.38	[1.33, 1.43]	<0.001	1.31	[1.27, 1.36]
2	Timed/ Timed	vs	Open/ Timed	1.13	[1.10, 1.17]	<0.001	1.08	[1.05, 1.12]
3	Timed/ Open	vs	Open/ Timed	1.08	[1.04, 1.11]	<0.001	1.04	[1.01, 1.07]
4	Timed/ Timed	vs	Timed/ Open	1.05	[1.02, 1.08]	0.002	1.04	[1.01, 1.07]
5	Open/ Timed	vs	Open/ Open	1.22	[1.17, 1.27]	<0.001	1.19	[1.15, 1.24]
6^a^	Timed/ Open	vs	Open/ Open	1.31	[1.27, 1.37]	<0.001	1.24	[1.20, 1.28]

If the IRR or RR < 1, the attendance rate was decreased for the considered invitation group compared to the reference category. If the IRR or RR = 1, no difference between the attendance rate of the considered invitation group and the reference category was observed. If the IRR or RR > 1, the attendance rate was increased for the considered invitation group compared to the reference category.

*IRR* incidence-rate ratio, *CI* confidence interval, *RR* relative risk.

^a^Additional analysis to the SAP.

Supplementary Fig. 1 explores women’s attendance over time. It shows that women who received timed appointments as their first invitation attended sooner in calendar time; and that timed second invitations also boosted attendance quite rapidly.

The same trend of increasing attendance with more timed invitations was broadly consistent across subgroups (Table 4). The two most deprived subgroups had the lowest attendance for all groups ranging from 41.1% (Open/Open; 95%CI 38.2–44.1%) to 63.4% (Timed/Timed; 95% CI 60.6–66.2%) and 40.2% (Open/Open; 95% CI 37.2–43.1%) to 61.5% (Timed/Timed; 95% CI 58.6–64.4%) for IMD 1 and 2 respectively. Timed/Timed resulted in attendance above 70% in the least deprived groups with attendance of 74% (95% CI 71.2–77.0%) and 78% (95% CI 74–81.4%) for IMD quintiles 4 and 5 respectively. The difference in attendance between Open/Open and Timed/Timed was larger in more deprived areas with an increase of 22.3% in IMD quintile 1 compared to 16.6% in IMD 5. Across services, attendance was lowest for Central & East London and North-East London, which remained below 60% even with two timed invitations (51.4%; 95% CI 43.1–59.7% and 57%; 95% CI 48.8–65.5% respectively; Supplementary Fig. 2). By contrast, Outer North-East London, North and East Devon, Cambridge and Doncaster all achieved attendance above 70% with at least one timed invitation. The absolute increase observed for Timed/Timed vs Open/Open ranged from approximately 15% in North London to ~22% in Dudley, Wolverhampton and SW Staffordshire. Area level estimates of ethnic diversity show that the same trends were observed in areas with different levels of ethnic diversity. First time invitees or previous never attenders (prevalent screen women) aged <60 y had the lowest 90-day attendance compared to other groups (33.0% (95% CI 29.3–36.7) to 54.7% (95%CI 49.0–60.4%) from Open/Open to Timed/Timed), but a large absolute increase in attendance (22%) between purely open and timed invitation strategies. The same trends were observed for the 180-day attendance (Table 4).

**Table 4:** Primary and secondary endpoint by subgroups.

Subgroup	Study arm	Total invited	90-day attendance	90-day attendance (%)	95%CI	180-day attendance	180-day attendance (%)	95%CI
***IMD quintile***								
**1: most deprived**	Open/ Open	1070	440	41.1%	[38.2%,44.1%]	489	45.70%	[42.7%,48.7%]
	Open/ Timed	1049	573	54.6%	[51.6%,57.6%]	609	58.06%	[55.1%,61.0%]
	Timed/ Open	1079	617	57.2%	[54.2%,60.1%]	632	58.57%	[55.6%,61.5%]
	Timed/ Timed	1115	707	63.4%	[60.6%,66.2%]	717	64.30%	[61.5%,67.1%]
**2**	Open/ Open	1041	418	40.2%	[37.2%,43.1%]	454	43.61%	[40.6%,46.6%]
	Open/ Timed	1102	594	53.9%	[51.0%,56.8%]	623	56.53%	[53.6%,59.5%]
	Timed/ Open	1147	681	59.4%	[56.5%,62.2%]	692	60.33%	[57.5%,63.2%]
	Timed/ Timed	1077	662	61.5%	[58.6%,64.4%]	674	62.58%	[59.7%,65.5%]
**3**	Open/ Open	776	408	52.6%	[49.1%,56.1%]	439	56.57%	[53.1%,60.1%]
	Open/ Timed	876	528	60.3%	[57.0%,63.5%]	568	64.84%	[61.7%,68.0%]
	Timed/ Open	809	538	66.5%	[63.2%,69.8%]	552	68.23%	[65.0%,71.4%]
	Timed/ Timed	828	563	68.0%	[64.8%,71.2%]	568	68.60%	[65.4%,71.8%]
**4**	Open/ Open	813	478	58.8%	[55.4%,62.2%]	517	63.59%	[60.3%,66.9%]
	Open/ Timed	839	567	67.6%	[64.4%,70.7%]	594	70.80%	[67.7%,73.9%]
	Timed/ Open	850	613	72.1%	[69.1%,75.1%]	622	73.18%	[70.2%,76.2%]
	Timed/ Timed	861	638	74.1%	[71.2%,77.0%]	650	75.49%	[72.6%,78.4%]
**5: least deprived**	Open/ Open	619	380	61.4%	[57.6%,65.2%]	403	65.11%	[61.4%,68.9%]
	Open/ Timed	555	387	69.7%	[65.9%,73.6%]	410	73.87%	[70.2%,77.5%]
	Timed/ Open	604	455	75.3%	[71.9%,78.8%]	466	77.15%	[73.8%,80.5%]
	Timed/ Timed	558	435	78.0%	[74.5%,81.4%]	439	78.67%	[75.3%,82.1%]
**Missing**	Open/ Open	38	17	44.7%	[28.9%,60.5%]	19	50.00%	[34.1%,65.9%]
	Open/ Timed	24	18	75.0%	[57.7%,92.3%]	18	75.00%	[57.7%,92.3%]
	Timed/ Open	36	24	66.7%	[51.3%,82.1%]	25	69.44%	[54.4%,84.5%]
	Timed/ Timed	36	32	88.9%	[78.6%,99.2%]	32	88.89%	[78.6%,99.2%]
***Prevalence and Age***								
**Prevalent and** < **60**	Open/ Open	1397	461	33.0%	[29.3%,36.7%]	517	37.0%	[33.0%,41.0%]
	Open/ Timed	1371	610	44.5%	[39.8%,49.2%]	659	48.1%	[43.0%,53.2%]
	Timed/ Open	1440	747	51.9%	[46.5%,57.2%]	770	53.5%	[47.9%,59.0%]
	Timed/ Timed	1411	772	54.7%	[49.0%,60.4%]	788	55.8%	[50.0%,61.7%]
**Incident and** < **60**	Open/ Open	1058	663	62.7%	[54.9%,70.5%]	722	68.2%	[59.4%,77.1%]
	Open/ Timed	1081	766	70.9%	[61.6%,80.2%]	812	75.1%	[64.8%,85.5%]
	Timed/ Open	1097	854	77.8%	[66.8%,88.9%]	873	79.6%	[67.9%,91.3%]
	Timed/ Timed	1104	886	80.3%	[68.4%,92.1%]	898	81.3%	[69.0%,93.7%]
**60** +	Open/ Open	1893	1017	53.7%	[48.9%,58.6%]	1,082	57.2%	[52.0%,62.4%]
	Open/ Timed	1979	1291	65.2%	[59.2%,71.3%]	1,351	68.3%	[61.8%,74.7%]
	Timed/ Open	1985	1327	66.9%	[60.6%,73.1%]	1,346	67.8%	[61.4%,74.2%]
	Timed/ Timed	1955	1379	70.5%	[63.7%,77.4%]	1,394	71.3%	[64.3%,78.3%]
***Service***								
**Doncaster**	Open/ Open	673	416	61.8%	[52.2%,71.4%]	446	66.3%	[55.7%,76.9%]
	Open/ Timed	664	488	73.5%	[60.8%,86.2%]	506	76.2%	[62.6%,89.8%]
	Timed/ Open	687	530	77.1%	[63.4%,90.9%]	540	78.6%	[64.3%,92.9%]
	Timed/ Timed	686	549	80.0%	[65.0%,95.0%]	554	80.8%	[65.4%,96.1%]
**Cambridge**	Open/ Open	561	309	55.1%	[45.9%,64.2%]	337	60.1%	[49.9%,70.2%]
	Open/ Timed	552	335	60.7%	[50.3%,71.1%]	366	66.3%	[54.6%,78.0%]
	Timed/ Open	595	432	72.6%	[59.5%,85.7%]	438	73.6%	[60.2%,87.0%]
	Timed/ Timed	593	429	72.3%	[59.3%,85.4%]	435	73.4%	[60.0%,86.7%]
**North London**	Open/ Open	717	297	41.4%	[35.3%,47.6%]	316	44.1%	[37.6%,50.6%]
	Open/ Timed	795	410	51.6%	[44.4%,58.7%]	422	53.1%	[45.7%,60.5%]
	Timed/ Open	762	396	52.0%	[44.6%,59.4%]	404	53.0%	[45.5%,60.6%]
	Timed/ Timed	733	419	57.2%	[48.8%,65.5%]	425	58.0%	[49.5%,66.5%]
**Outer NE London**	Open/ Open	404	214	53.0%	[42.6%,63.3%]	221	54.7%	[44.0%,65.4%]
	Open/ Timed	403	282	70.0%	[55.1%,84.9%]	287	71.2%	[55.9%,86.6%]
	Timed/ Open	407	268	65.8%	[52.4%,79.3%]	276	67.8%	[53.7%,81.9%]
	Timed/ Timed	451	327	72.5%	[57.5%,87.5%]	330	73.2%	[57.9%,88.4%]
**Central and East London**	Open/ Open	564	182	32.3%	[26.6%,38.0%]	206	36.5%	[30.3%,42.8%]
	Open/ Timed	583	280	48.0%	[40.2%,55.8%]	300	51.5%	[43.1%,59.8%]
	Timed/ Open	588	294	50.0%	[41.9%,58.1%]	303	51.5%	[43.2%,59.9%]
	Timed/ Timed	591	304	51.4%	[43.1%,59.7%]	311	52.6%	[44.1%,61.1%]
**North and East Devon**	Open/ Open	612	340	55.6%	[46.7%,64.4%]	355	58.0%	[48.7%,67.3%]
	Open/ Timed	640	417	65.2%	[54.6%,75.8%]	430	67.2%	[56.1%,78.3%]
	Timed/ Open	614	447	72.8%	[59.9%,85.7%]	455	74.1%	[60.7%,87.5%]
	Timed/ Timed	602	448	74.4%	[60.8%,88.0%]	455	75.6%	[61.5%,89.6%]
**Dudley, Wolverh and SW Staffords**^**a**^	Open/ Open	826	383	46.4%	[40.0%,52.7%]	440	53.3%	[46.0%,60.5%]
	Open/ Timed	808	455	56.3%	[48.5%,64.1%]	511	63.2%	[54.2%,72.3%]
	Timed/ Open	872	561	64.3%	[55.4%,73.2%]	573	65.7%	[56.5%,74.9%]
	Timed/ Timed	819	561	68.5%	[58.4%,78.6%]	570	69.6%	[59.2%,80.0%]
**Ethnicity**^**b**^ **(area level estimate)**								
**White**	Open/ Open			51.0%	[47.4%, 54.7%]		55.3%	
	Open/ Timed			62.6%	[58.1%, 67.2%]		66.2%	
	Timed/ Open			67.3%	[62.3%, 72.3%]		68.7%	
	Timed/ Timed			71.0%	[65.5%, 76.5%]		72.0%	
**Non-white**	Open/ Open			43.4%	[37.9%, 48.8%]		47.1%	
	Open/ Timed			52.2%	[45.8%, 58.5%]		55.3%	
	Timed/ Open			56.8%	[49.9%, 63.8%]		58.1%	
	Timed/ Timed			58.5%	[51.4%, 65.7%]		59.5%	

^a^Dudley, Wolverhampton, and Southwest Staffordshire.

^b^Ethnicity calculation excludes the missing category. See supplementary for further details. Non-white includes ethnicity Asian, Black, Mixed and Other, excludes women with LSOA linkage to her postcode.

## Discussion

This service evaluation was, to our knowledge, the first prospective investigation of different combinations of timed appointments vs open invitations for initial breast screening invitations and second invitations for non-attenders. A Timed/Timed invitation approach was statistically superior to all other strategies. Having at least one timed appointment improved attendance when compared to only open invitations. When only sending one timed invitation, an initial timed invitation was better than an open invitation followed by a timed second invitation. Use of only open invitations did not achieve the national benchmark of 70% attendance overall or in any subgroup. Similar effects were observed in all subgroups, but the absolute uptake varied. The impact of using Timed/Timed invitations was the greatest in the two most deprived quintiles, where the absolute difference in uptake between Open/Open and Timed/Timed was over 20%. The Timed/Timed approach might therefore reduce socio-economic inequalities in uptake.

There is consistent evidence that women experiencing socioeconomic deprivation are less likely to attend breast screening in England, across Europe, and in Canada, South Korea, Israel and Australia [10]. The NHSBSP and charities like Cancer Research UK and Breast Cancer Now have set out strategies to reduce inequalities in screening [11, 12]. Interventions that aim to increase uptake are an important approach for addressing social inequalities in cancer outcomes. A recent meta-analysis found that four interventions resulted in higher screening uptake in the most deprived vs least deprived areas in randomised controlled trials (RCTs) for screening programmes or tests other than breast [13]: multilingual approaches by phone, follow-up calls, reminder interventions and ‘implementation intention’ interventions (leaflets with goal-directed plans promoting screening). However, this review did not identify any RCTs of interventions that had been shown to be more effective in more deprived compared to less deprived areas for breast screening. Our finding that Timed/Timed invitations were more effective in more deprived areas shows that this strategy is unique as a population level intervention with the potential to reduce inequalities in breast screening uptake across England. By contrast, the other interventions recommended in the guidance mentioned above are mostly aimed at local populations. For example, removing logistical barriers locally has been used to reduce inequalities. For example, mobile mammography units, which have been routinely used in many countries including England for six decades, reduce logistical barriers by moving the units closer to areas where women live thereby reducing travel time to their appointments [11, 14]. Moving a mobile unit to a new and more convenient location in a deprived area in Dudley in the UK, in combination with a targeted campaign, recently had a positive impact on uptake [15]. A qualitative study in the US showed that, amongst African American women, the use of mobile units was perceived as an effective strategy to increase attendance [16].

Difficulty making a convenient appointment has frequently been recorded as a barrier in other studies [17]. Providing women with an appointment time and date removes the additional step of having to call to book an appointment. This potentially removes some logistical barriers to participation and has been cited as a reason why breast screening is less easy to postpone or forget than home-based colorectal screening [18]. This reasoning resonates with results from another part of this service evaluation, where a survey was conducted in non-attenders [19]. Here, approximately 30% of those non-attenders who responded to the survey reported issues making a convenient appointment, and 25% reported having too many other things to worry about.

While invitations with timed appointments increased attendance, they did not yield attendance above 70% for women in more deprived and ethnically diverse areas in our evaluation. Culture and language sensitive telephone reminders have been shown to increase screening attendance in these areas, for example in North East London and elsewhere [13, 20]. For these interventions, women from the same cultural background as the majority of local women, therefore understanding their cultural barriers and speaking their languages, phone non-attenders to answer questions and make appointments. Other interventions that might be helpful are decisions aids [13], for example a newly developed animated video, which is currently being trialled [21].

Three RCTs have investigated the use of timed or pre-scheduled appointment letters on mammography uptake [7, 22, 23]. One tested the effect of different invitation systems or letters on all women eligible for screening and found that using timed (initial) invitation letters significantly increased attendance at breast screening compared to open invitations [22]. Two RCTs specifically looked at timed appointments for non-attenders and found that timed appointments increased attendance for women with a history of non- or irregular attendance or current non-attenders [7, 23]. These RCTs reported absolute increases in attendance ranging between 4 and 21% for timed appointments, where the 21% increase occurred when timed appointments were combined with primary care endorsement. Furthermore, during the Covid recovery period, two studies observed a 7% higher attendance for first timed appointments compared to open invitations in London and in South England [8, 24]. Although none of these studies assessed the impact of open invitations vs timed appointment for combinations of first and second invitations, the magnitude of the increase in uptake was in line with the findings from our evaluation. We observed an absolute increase in attendance between 3 and 19%. The highest increase (19% for Timed/Timed) was similar to the 21% observed by Segnan et al. [22], when two interventions were combined.

This service evaluation was conducted in routine practice, using invitation strategies currently being used in practice. Therefore, findings are likely to be generalisable and suitable to inform national recommendations for the English screening programme. The evaluation used a sufficiently large sample size for important subgroup comparisons of different invitation strategies, including by IMD quintile and screening service. Including screening services in areas with higher socioeconomic deprivation and ones with historically lower uptake allowed for sufficient uptake to understand the impact of different invitation strategies among underserved population. The evaluation also included services located in different parts of England and catchment areas varying in rurality. One important limitation is that it was not possible to obtain ethnicity data for individual women, and therefore the findings reported on ethnicity subgroups are at risk of bias due to the assumptions used to estimate effects by ethnic group. In particular, they are based on assuming the ethnicity of those invited was proportionally the same as LSOA-level information reported in the 2021 census, and the attendance rate within each LSOA was the same for each ethnic group. In other words, it is possible that the pattern by invitation strategy reported between ethnic groups in our analysis may not reflect the true pattern; further service evaluations might consider this issue.

## Conclusion

This evaluation confirmed that the use of timed appointments results in the higher attendance. Importantly, we showed for the first time that timed appointments in first and second invitations in the NHSBSP, where feasible, should result in higher attendance than any strategy including open invitations. Furthermore, the impact of timed compared with open invitations appears to be greater in areas of higher deprivation. Therefore, use of timed appointments might also help improve health equity by reducing social inequalities arising from differences in screening uptake. The results from this evaluation provided a robust evidence-base to support a change in national service specifications for NHSE on the most effective invitation methodologies for services to move to from April 2025.

## Supplementary information

Supplementary Materials

## Data Availability

Individual anonymised participant data are held by QMUL under a data sharing agreement with NHSE, and any requests for individual participant data should be addressed to NHSE.
