# Supplementary material for: The effect of different combinations of open invitations and timed appointments on breast screening attendance: service evaluation of invitation strategies in the NHS Breast Screening Programme
Source: Br J Cancer. 2026 May 14;135(4):630–40. doi: 10.1038/s41416-026-03436-8 (PMC13427739; doi:10.1038/s41416-026-03436-8)
Supplement: Supplementary file 1 — Supplementary Materials [file 41416_2026_3436_MOESM1_ESM.docx]

**Supplementary materials**

**Additional statistical methods**

*Estimating ethnicity by LSOA*

The data and analytical team in NHS England supplied the LSOA code based on the residential postcode of most women involved in this study. The estimated proportion of an ethnicity in each arm or service is calculated by weighing three elements and summing across LSOAs. First the composition of different LSOA in each arm or service, second the composition of different ethnicity in each LSOA, third the proportion of an arm or a service in the study.

For example, there were 48 women in this study from LSOA code E01028027, of which 13 (27%) were assigned to Timed/ Timed invitation strategy. According to census data,^1^ 66% women from this LSOA between age 50 and 74 years are of white ethnicity. We estimated (48 x 27% x 66% =) 8.54 women were allocated to Timed/Timed from LSOA E01028027 who are white. Sum this number across all LSOAs in a given arm will give the total number of women allocated to this arm in each ethnicity.

The definition of white and non-white adopted from the *Ethnic group classification 6a used in Census 2021*.^2^ Non-white incudes Asian, Black, Mixed and Other ethnic group, we had no women in the “does not apply” group, some women had missing LSOA linkage to the postcode from NHS Data Team and recorded as missing, excluded from the non-white ethnic group.

*Estimating attendance in each ethnicity by invitation strategy*

This is estimated using the LSOA code based on the residential postcode of most women involved in this study. Due to limitation of dataset, we could not estimate the probability for a woman being white for when she has attended in a given arm. As a result, estimate presented slightly underestimate attendance for the subgroup Timed/Timed and white, and overestimate for the subgroup Open/Open and non-white. The number of women who have attended within 90 days (180 days) who are white and assigned to Open/Open is estimated by:

Total number of women with LSOA recorded multiply by the probability of being assigned to Open/Open arm, then multiply by the probability of attend within 90 (180 days) using the assigned strategy, finally multiply by the probability of the women being white if she has attended.

*Use of letter print date when observing attendance over time*

When looking at the 90- and 180-day attendance over time amongst 4 invitation strategies since the initial appointment, we used 120- and 210-day attendance since the first invitation letter was printed, instead of using the period since the first appointment was offered. This was to account for the fact when women receive an open invitation, she can book an appointment and therefore attend earlier than the date originally assigned to the virtual clinic. i.e. virtual clinics tend to be 4 weeks since the letter sent, and a woman could call to have an appointment the next day from when the letter was received. When a letter was printed, it was expected to be sent the same or the next day and should be received within a week. As the date printed was around 30 days earlier than a virtual clinic could have been, this was added to 90 and 180 days when date of letter printed was used as the starting point for looking at attendance over time.

**Supplementary Tables**

**Supplementary Table 1**: Breast screening uptake in 2021/22 and Index of Multiple Deprivation (IMD) quintile of selected services determined by the NHSBSP prior to the start of the study

| **Code** | **Service** | **Uptake^a^** | **IMD Quintile^b^** |
| --- | --- | --- | --- |
| FLO | Central and East London | 42% | 3 |
| EBA | North London | 54% | 2 |
| FBH | Outer NE London | 58% | 1 |
| MDU | Dudley, Wolverh. & SW Staffords. | 60% | 2 |
| DCB | Cambridge | 61% | 5 |
| CDO | Doncaster | 63% | 2 |
| LED | North and East Devon | 67% | 4 |
| ^a^ Year 2021/22 figure^3^  ^b^ 1 being the most deprived and 5 being the least deprived.. | | | |

**Supplementary Table 2**: Characteristics of women included in the services evaluation by NHSBSP service.

|  | Service name | | | | | | | | | | | | | |
| --- | --- | --- | --- | --- | --- | --- | --- | --- | --- | --- | --- | --- | --- | --- |
|  | Doncaster | | Cambridge | | North London | | Outer NE London | | Central and East London | | North and East Devon | | Dudley,Wolverh&  SW Staffords ^d^ | |
| Age (Mean, IQR) ^a^ | 60.0 | 54.0 – 65.0 | 54.4 | 50,0-59.0 | 59.4 | 55.0-64.0 | 58.8 | 54.0-64.0 | 58.5 | 54.0.63.0 | 59.1 | 55.0-65.0 | 58.4 | 52.0-64.0 |
|  |  |  |  |  |  |  |  |  |  |  |  |  |  |  |
| IMD (quintile) |  |  |  |  |  |  |  |  |  |  |  |  |  |  |
| 1: most deprived | 628.0 | (23.2%) | 83.0 | (3.6%) | 382.0 | (12.7%) | 1018.0 | (61.1%) | 514.0 | (22.1%) | 199.0 | (8.1%) | 1489.0 | (44.8%) |
| 2 | 422.0 | (15.6%) | 91.0 | (4.0%) | 780.0 | (25.9%) | 619.0 | (37.2%) | 1346.0 | (57.9%) | 306.0 | (12.4%) | 803.0 | (24.2%) |
| 3 | 564.0 | (20.8%) | 356.0 | (15.5%) | 743.0 | (24.7%) | 22.0 | (1.3%) | 304.0 | (13.1%) | 775.0 | (31.4%) | 525.0 | (15.8%) |
| 4 | 719.0 | (26.5%) | 679.0 | (29.5%) | 796.0 | (26.5%) | 2.0 | (0.1%) | 125.0 | (5.4%) | 616.0 | (25.0%) | 426.0 | (12.8%) |
| 5: least deprived | 359.0 | (13.2%) | 1051.0 | (45.7%) | 285.0 | (9.5%) | 0.0 | (0.0%) | 26.0 | (1.1%) | 542.0 | (22.0%) | 73.0 | (2.2%) |
| missing | 18.0 | (0.7%) | 41.0 | (1.8%) | 21.0 | (0.7%) | 4.0 | (0.2%) | 11.0 | (0.5%) | 30.0 | (1.2%) | 9.0 | (0.3%) |
|  |  |  |  |  |  |  |  |  |  |  |  |  |  |  |
| Prevalence and age |  |  |  |  |  |  |  |  |  |  |  |  |  |  |
| prevalent and <60 | 494.0 | (18.2%) | 1556.0 | (67.6%) | 762.0 | (25.3%) | 356.0 | (21.4%) | 805.0 | (34.6%) | 631.0 | (25.6%) | 1015.0 | (30.5%) |
| incident and <60 | 771.0 | (28.5%) | 190.0 | (8.3%) | 821.0 | (27.3%) | 570.0 | (34.2%) | 545.0 | (23.4%) | 616.0 | (25.0%) | 827.0 | (24.9%) |
| 60+ | 1445.0 | (53.3%) | 531.0 | (23.1%) | 1424.0 | (47.4%) | 734.0 | (44.1%) | 974.0 | (41.9%) | 1221.0 | (49.5%) | 1483.0 | (44.6%) |
| missing | 0.0 | (0.0%) | 24.0 | (1.0%) | 0.0 | (0.0%) | 5.0 | (0.3%) | 2.0 | (0.1%) | 0.0 | (0.0%) | 0.0 | (0.0%) |
|  |  |  |  |  |  |  |  |  |  |  |  |  |  |  |
| Ethnicity ^b^ |  |  |  |  |  |  |  |  |  |  |  |  |  |  |
| Asian | 37.5 | (1.4%) | 71.1 | (3.1%) | 619.4 | (20.6%) | 143.0 | (8.6%) | 305.7 | (13.1%) | 37.8 | (1.5%) | 685.1 | (20.6%) |
| Black | 13.6 | (0.5%) | 22.7 | (1.0%) | 407.2 | (13.5%) | 331.8 | (19.9%) | 398.1 | (17.1%) | 7.8 | (0.3%) | 218.4 | (6.6%) |
| Mixed | 11.2 | (0.4%) | 17.5 | (0.8%) | 73.5 | (2.4%) | 21.5 | (1.3%) | 62.0 | (2.7%) | 15.0 | (0.6%) | 43.2 | (1.3%) |
| White | 2437.8 | (90.0%) | 1898.5 | (82.5%) | 1485.8 | (49.4%) | 1073.0 | (64.4%) | 1263.4 | (54.3%) | 2054.5 | (83.2%) | 2183.4 | (65.7%) |
| Other | 8.0 | (0.3%) | 14.3 | (0.6%) | 272.1 | (9.0%) | 31.7 | (1.9%) | 166.7 | (7.2%) | 11.8 | (0.5%) | 91.9 | (2.8%) |
| Missing ^c^ | 202.0 | (7.5%) | 277.0 | (12.0%) | 149.0 | (5.0%) | 64.0 | (3.8%) | 130.0 | (5.6%) | 341.0 | (13.8%) | 103.0 | (3.1%) |
| IMD, index of multiple deprivation; IQR, interquartile range   1. Age in years at first offered appointment. 2. Ethnicity is estimated based on the LSOA of women’s residential postcode, not of individual woman. See [appendix](#_Estimating_ethnicity_by) for further detail. 3. Women whose LSOA was unable to be linked to identify residential region. 4. Dudley, Wolverhampton, and Southwest Staffordshire. | | | | | | | | | | | | | | |

(a)


(b)

**Supplementary Figure 1** Proportion of women attending within 120 (a) and 210 (b) days since letter printed by invitation strategy. The red dotted line indicates the 70% uptake target as set by the NHS breast screening programme.


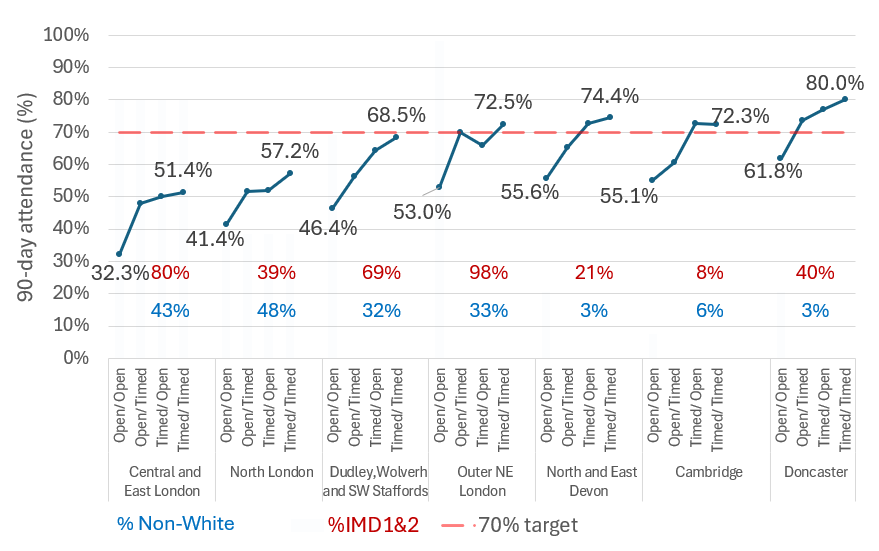


**Supplementary Figure 2**: Proportion of 90-day attendance by service and invitation strategy. Percentage non-White was an estimate based on area-level ethnicity. The Lower Super Output Area (LSOA) code linked to the residential postcode of each woman in the study was used to estimate the proportion of each ethnic group within each screening service. Estimates were derived by weighting and summing across LSOAs using three components: (1) the distribution of LSOAs within each service, (2) the ethnic composition of each LSOA, and (3) the proportion of the study population contributed by each service. IMD scores were obtained at the LSOA level (English Indices of Deprivation 2019) and weighted using GP practice registration counts before mapping to screening centres.

**List of changes to the Statistical Analysis Plan**

Update on 10^th^ April 2024

1. The data were planned to be followed until the 31^st^ of January 2024 and be analysed in February and March. In practice, further follow-up was obtained till the 19^th^ of April, and the timing of analysis was till the 3^rd^ of May 2024.

**Statistical Analysis Plan for**

**Invitation Strategy Evaluation**

**An Evaluation of the Effect of Open Invitation and Timed Appointments on Attendance at the NHS Breast Screening Programme**

| **Version Number** | 1.0 |
| --- | --- |
| **Effective Date** | 15/01/2024 |

| **Project Statistician** | Joy Li  Name | Statistician |
| --- | --- | --- |
|  | 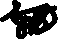 Signature | Date  23/01/2024 |
| **Approved by** | Stephen Duffy  Name | Chief Investigator |
|  | **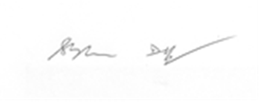**  Signature | Date  23/01/2024 |

| **Version** | **Date** | **Reason for Change** |
| --- | --- | --- |
| 1.0 | 23/01/2024 | N/A |
| 1.1 | 22/02/2024 | 1. Service in East Lancashire did not participate at the end, so exclude from all tables and figures to be produced. 2. After data is extracted, we realise there is no information on whether a woman has participated in the previous episode, so to be excluded from tables. 3. Exclusion regarding special requirement, only ones with learning disability to be excluded after confirming with JC |
|  |  |  |
|  |  |  |
|  |  |  |

Contents

[1 Introduction 5](#_Toc155961859)

[2 Study details 5](#_Toc155961860)

[2.1 Objectives 5](#_Toc155961861)

[2.2 Design 5](#_Toc155961862)

[2.3 Setting 5](#_Toc155961863)

[2.4 Inclusion / exclusion criteria 6](#_Toc155961864)

[2.5 Follow-up 6](#_Toc155961865)

[2.6 Primary Endpoint 6](#_Toc155961866)

[2.7 Secondary Endpoints 6](#_Toc155961867)

[2.8 Subgroups 6](#_Toc155961868)

[2.9 Sample Size Justification 8](#_Toc155961869)

[2.10 Timing of analysis 8](#_Toc155961870)

[3 Study data 8](#_Toc155961871)

[3.1 Preparing data for analysis 8](#_Toc155961872)

[3.2 Variables for analysis 9](#_Toc155961873)

[3.2.1 From source data 9](#_Toc155961874)

[3.2.2 Data linkage and variable derivation 10](#_Toc155961875)

[4 Statistical Analysis 12](#_Toc155961876)

[4.1 General principles 12](#_Toc155961877)

[4.2 Primary analysis 13](#_Toc155961878)

[4.3 Secondary analysis 13](#_Toc155961879)

[4.4 Primary endpoint methods 13](#_Toc155961880)

[4.5 Secondary endpoint methods 14](#_Toc155961881)

[4.6 Subgroup analysis 14](#_Toc155961882)

[4.7 Other analysis 14](#_Toc155961883)

[5 Presentation of analysis 14](#_Toc155961884)

[5.1 Tables 15](#_Toc155961885)

[5.2 Figures 15](#_Toc155961886)

[6 References 16](#_Toc155961887)

[7 Appendices 17](#_Toc155961888)

[7.1 Data dictionary 17](#_Toc155961889)

[7.2 Template for tables and figure 19](#_Toc155961890)

# Introduction

Attendance in the UK NHS Breast Screening Programme has previously exceeded 70% on average. In the past, women initially received a timed appointment as their first invitation, followed by either a 2^nd^ timed appointment or an open invitation for women who did not attend their first appointment. Previous work showed that a second timed appointment letter was effective at improving screening participation^1^. However, during the Covid pandemic open invitation letters were used routinely to help the screening services to recover more quickly^2^. With the NHS breast screening services (NHSBSP) moving to a new IT system, open invitation letters with online and phone booking are planned to be used as standard from 2024/25.

This study has been commissioned to evaluate the effect of different combinations of timed appointments and open invitations on attendance rate at the NHS Breast Screening Programme. Below describes the statistical analysis plan for the evaluation of the effect of different strategies, either timed or open appointments, for the first invitation, or second invitation for non-attenders.

# Study details

## Objectives

The primary objectives are to evaluate:

1. the effect of different combinations of timed appointments and open invitations on attendance in the NHS Breast Screening Programme; and

2. the effect as above in subgroups defined by index of multiple deprivation (IMD), and area-level summaries of ethnic diversity.

## Design

To meet these objectives, an evaluation was run to compare four strategies:

1. Timed for both first and second invitation (TT),
2. Open invitations for both (OO),
3. Timed followed by Open invitation (TO), and
4. Open followed by Timed invitations (OT).

Women were allocated to each strategy based on whether certain digits in their screening identifier (SX) and NHS number were even or odd (quasi-randomisation as shown in figure 1 of the evaluation protocol for randomisation procedure).

## Setting

The evaluation took place in 8 national Breast Screening Program services:

1. Central and East London (pilot area where the study was first run) (FLO),
2. Outer Northeast London (FBH),
3. North London (EBA),
4. Cambridge (DCB),
5. North and East Devon (LED),
6. Doncaster (CDO),
7. Dudley, Wolverhampton, and Southwest Staffordshire (MDU), and

These services were selected to ensure socio-economic and cultural diversity and included several areas with lower attendance than average in England.

## Inclusion / exclusion criteria

Women within the above services were eligible for this evaluation if they were due to be invited for their next breast screening appointment during the study period. Exclusions were made based on five criteria (as of section 5.2 of the evaluation protocol):

1. Opted out of the screening programme.
2. Require special appointments that require different types of clinics.
3. Self-referred for screening.
4. Women on an early recall protocol.
5. Women who are invited because they are high risk.

## Follow-up

Attendance assessed from date of invitation until the end of January 2024 (data extraction date).

## Primary Endpoint

The 90-day attendance rate. This is defined as attending the originally offered screening appointment, or an alternative appointment within 90 days of the original. For open invitations, the originally offered date is the date of the holding clinic, into which women were booked at the time of sending the first open invitation letter.

## Secondary Endpoints

The 180-day attendance rate, defined as attending the originally offered screening appointment, or an alternative appointment within 180 days of the original. For open invitations, the originally offered date is the date of the initial holding clinic, into which women were booked at the time of sending the first open invitation letter.

## Subgroups

Subgroups for reporting are below, further details are in [section 3.2.1](#source_data_variable) and [section 3.2.2](#derived_variables):

1. IMD (quintile):
   - 1: most deprived
   - 2
   - 3
   - 4
   - 5: least deprived
2. Ethnicity(area-level):

- White
- Non-white

1. Combination of prevalent/incidence screen and binary age categories:
   - Prevalent and under 60
   - Incident and under 60
   - Age 60 and over
2. Screening services:
   - Central and East London (pilot area where the study was first run)
   - Outer Northeast London
   - North London
   - Cambridge
   - North and East Devon
   - Doncaster
   - Dudley, Wolverhampton, and Southwest Staffordshire
   - East Lancashire
3. Rurality of the screening site:
   - Rural
   - Urban
4. Mobility of the screening clinic:
   - Static, e.g. hospital
   - Mobile, e.g. van

Our focus lies in the first two subgroups, IMD quintiles and the area-level ethnicity.

## Sample Size Justification

We aimed for a sample size of 16,000 women for the following reasons.

Previous results comparing timed to open invitations suggest an absolute 8% increase in participation with timed appointments compared to open invitations. As noted above, there is an interest in subgroup analyses. For a comparison of two groups with participation rates of 65% and 73%, 524 persons per group would give 80% power to detect this difference as significant at 5% level with two-sided testing. To allow for estimation of the primary outcome in socioeconomic and ethnic subgroups, we planned to evaluate 16,000 women in total (4,000 per arm).

## Timing of analysis

Data will be analysed in February and March 2024. The statistical analysis will be performed at the Centre for Cancer Screening, Prevention and Detection, Wolfson Institute of Population Health, Barts and The London School of Medicine and Dentistry, Queen Mary University of London.

# Study data

A list of variables will be extracted according to the study protocol, the full list is presented in [section 7.3](#list_all_vars) in the appendices.

* In this document, names of variables will be in *italic* format.

## Preparing data for analysis

- The source dataset from each service has the same format and variables. To prepare a master file of all services the separate extracts must be joined together, with a new file to indicate which service each extract was from.

STATA command **Append** and **generate** a new variable *service* to indicate the source screening service.

- The joined dataset has each line representing one appointment, not per woman. To restructure the data so that each line represents a woman.

STATA command **reshape** from long to wide format using a unique identifier variable (*uniqueid)* and a variable tag each appointment (*[appointmenttag](#appointmenttag_create)*).

Record the total number of women in the final sample. ^1^

- Exclude all screenees who has special requirement based on [exclusion criteria](#inclusion_exclusion_criteria).

**Drop** if ‘Y’ in *specialrequirement.* Record the number of women in this group.^1^

- To assess for risk of bias later, ensure to flag all screenees with administrative errors, e.g. when a wrong type of invitation letter was sent (‘Y’ in *flag2oiinviteandnotaoicl* or in *flag3tabutanoiletterused)*, and keep a record of this number in each arm.^1^

## Variables for analysis

### From source data

The subset of variables from source dataset to be used for our analysis are:

| Variable | Definition | Notes |
| --- | --- | --- |
| *Lsoacode* | Lower Layer Super Output Areas | For various data linkage with census data for demographic profile. |
| *Uniqueid* | Unique patient ID | Generated internally by BSS in place of screening id, unique across the national dataset. |
| *Ageatfoa* | Age at first offered appointment in the screening episode | Range expected [48,70]. |
| *attendedappointment* | Whether an appointment has been attended in this episode | This indicates whether a woman attended her appointment but not whether she was screened. Sometimes women turn up and are not screened for reasons such as, they recently had a mammogram, or they decided not to be screened after arrival. |
| *Screeningdate* | Date of the screening | Empty if not screened. |
| *previousscreeningdate* | The date of previous screening | For incident screenees only. |
| *Armname* | Arm of the study | - 1: OI/OI, - 2: OI/TA, - 3: TA/OI, and - 4: TA/TA. |
| *prevalentincidentstatus* | Prevalent or incidence screening episode | - I: incident, episode complete, - P: prevalent, episode complete, - XI: incident, episode yet to complete, and - XP: prevalent, episode yet to complete. |
| *Date* | Date of the appointment offered/booked | For open invitation, this is an assumed appointment date in a holding clinic. |
| flag2oiinviteandnotaoicl | Flag for when an open invitation letter was used when it should not be | For assessing the risk of bias. Should mostly be missing. |
| flag3tabutanoiletterused | Flag for when a timed appointment letter was used when it should not be | For assessing the risk of bias. Should mostly be missing. |
| *Locationnature* | Whether the clinic was static (hospital) or mobile (van) |  |

### Data linkage and variable derivation

The following variables are derived based on the source data.

| Variable | Definition | Notes |
| --- | --- | --- |
| *service* | Screening service | - Derived from service of which each data was extracted from: - 1, Central and East London (pilot area). - 2, Outer Northeast London. - 3, North London. - 4, Cambridge. - 5, North and East Devon. - 6, Doncaster. - 7, Dudley, Wolverhampton and Southwest Staffordshire, and - 8, East Lancashire. |
| *appointmenttag* | Tag each appointment, in the chronological order of appointment date. | Derived from *date*, labelled 1, 2, 3, etc by the chorological order of each appointment offered/ booked.  For reshaping data between wide and long format, each line represents either an appointment or per woman. |
| *flagadminerror* | Flag for administrative errors that wrong type of invitation letter was sent. | Derived from *flag2oiinviteandnotaoicl* and *flag3tabutanoiletterused.*   - 1, if Y on either variable above, error. - 0, otherwise, no error.   Should be a very small proportion of the sample. |
| *90dayattend* | Primary endpoint, 90-day attendance since first offered appointment | Derived from *attendedappointment and date*.   - 1, if Y on *attendedappointment* and the difference between variable screeningdate and variable *date* is less than or equal to 90 days. - 0, if N on *attendedappointment*, or attended but the difference between variable screeningdate and variable date is greater than 90 days: |
| *180_survtime* | Follow-up duration | - If Y on *attendedappointment, then t*he number of days between variable *screeningdate* and variable *date* if it is less than or equal to 180 days. - Otherwise, then *t*he number of days between variable *date* and the 31^st^ of January 2024 or the end of 180-day follow-up, whichever is the first.   As the time of event/censoring time in cox regression. |
| *180dayattend* | Secondary endpoint, 180-day attendance since first offered appointment | - 1, if Y on *attendedappointment* and the difference between variable *screeningdate* and variable *date* is less than or equal to 180 days, or to the 31^st^ of January 2024, whichever is the first. - 0, if N on *attendedappointment*, or attended but the difference between variable screeningdate and variable date is greater than 180 days.   This is the event variable for cox regression. |
| *imdquintile* | Index of Multiple Deprivation in quintiles | First, small area level index of multiple deprivation 2019 (*imddecile*) by linking *lsoacode (*2011) with IMD2019 data^2^, then derive from deciles to quintiles.   - 1 (least deprived) - 2 - 3 - 4 - 5(most deprived) |
| *ethnicity* | ethnicity (area level estimate) | Small area level ethnicity estimate derived by linking source data with the ethnicity data from Office for National Statistics site^3^ with *lsoacode* as identifier, for women from aged 50 and above, by linking LSOA with census data. Expect in 5 major categories:   - - 1, Asian or Asian British   - 2, Black or Black British   - 3, Mixed   - 4, Other ethnic groups   - 5, White |
| *ethnicity_white* |  | - - Derived from *ethnicity* as defined above.   - 1, white   - 0, non-white |
| *rurality* | Whether the screening site is rural or urban. | Derived by linking source data with the data from open geography portal, on ‘Rural Urban Classification (2011) of Lower Layer Super Output Areas in England and Wales”^4^, using *lsoacode* as identifier.   - - 0, rural   - 1, urban |
| *age_prev_status* |  | Derived from *ageatfoa* and *prevalentincidentstatus:*   - - 1, prevalent and under60,   - 2, incident and under60,   - 3, over60, and   If *prevalentincidentstatus* is missing, impute based on *ageatfoa.* |
| *firstarm* | Open invitation or timed appointment at the first invite. | Derived from *armname* |
| *secondarm* | Open invitation or timed appointment at the second invite. | Derived from *armname* |
| Note: when the *lsoacode* cannot be identified in the sourcedate during data linkage, resolve from checking the data file: LSOA (2011) to LSOA (2021) to Local Authority District (2022) lookup for England and Wales. ^5^ | | |

**NOTE:** The original source dataset should not be altered. Any modification to data extract MUST be saved to a separate file.

# Statistical Analysis

## General principles

**Analysis data**: Women who fall in the [exclusion criteria](#inclusion_exclusion_criteria) will be excluded from all analyses.

**Quantifying uncertainty:** Unless stated otherwise all p-values will be two-sided, and 95% confidence intervals used. There will be no adjustment for multiple testing unless specified.

**Missing data**: Primary analysis will include all women within the inclusion criteria and with complete data on primary endpoint. Details for dealing with missingness when deriving variable are in [section 3.2.2.](#derived_variables)

Overall, very low level of missingness is expected for any variable. In case of missingness on source data, all enquires will be first consulted with data team of the programme, to check whether this can be completed. If the problem cannot be solved, data imputation will be used for a variable, only if the missingness exceeds 80 screenees (5% of the total expected study participants).

**Reporting:** Statistical significance will be assessed at the two-sided 5% level unless otherwise stated. 95% confidence intervals will be provided as appropriate.

**Software**: The data management and analysis will be carried out using computer programme STATA version 17.0^6^.

## Primary analysis

For the primary analysis a hierarchical approach will be used for testing at the 5% level. In the first instance, a global likelihood ratio test on 3 degrees of freedom will be applied to assess the significance of a difference in any direction among the groups overall. Also, a four-way comparison with OO as the reference category.

To account for multiple comparisons, null hypotheses (no difference in 90-day screening attendance between arms) will be tested in the following order:

(1) Timed Timed (TT) vs Open Open (OO).

(2) TT vs OT.

(3) TO vs OT.

(4) TT vs TO.

(5) OT vs OO.

Testing will only proceed if the preceding hypothesis was rejected at the 5% level (p-value <0.05).

Independently of significance test results, both overall and for specific subgroups, we will report the most successful group in terms of 90-day attendance.

## Secondary analysis

For the secondary analysis, we will use hierarchical approach to compare attendance for the two invitation strategies for

1. the first invite (TT+TO) vs (OT+OO) and
2. the second invite (TT+OT) vs (TO+OO).

Effect sizes will be presented with 95%CIs and p-value (Bonferroni corrected).

## Primary endpoint methods

For the primary endpoint, Poisson regression will be used, with number of subjects attending within 90 days of their first offered appointment as the outcome (*90dayattend)*, offset by total numbers invited.

The attendance rate within 90 days of the first offered appointment in the study arms is compared using Poisson regression model with robust variance estimates to take account of clustering in the allocation to arms OI and TA at the first invitation^7^. In STATA the command **poisson** with option **vce(robust)** is used, reporting with 95% confidence interval using **level (95)**, and with option **irr** to output estimated coefficient as incidence-rate ratios.

A likelihood-ratio test is used to check the significance of the regression coefficient in the poisson regression model (STATA **lrtest**). Statistical testing will be two-sided, with p<0.05 considered to be statistically significant. We will compare each of the other groups with the OO group as reference category. Thereafter pairwise comparisons will be carried out as described above. We conclude the following for

- IRR < 1, the attendance rate is decreased for the considered study arm compared to the reference category.
- IRR = 1, no difference between the attendance rate of the considered study arm and the reference category.
- IRR > 1, the attendance rate is increased for the considered study arm compared to the reference category.

The IRR is significantly greater or smaller than 1, if 1 is not within the border of the 95% CI, which is analogue to the p-value is smaller than 0.05.

## Secondary endpoint methods

The secondary endpoint, the attendance rate at 180 days since first offered, time-to-event analysis using Kaplan Meier (KM) survival analysis will be used. Data need to be first set up to carry out this analysis using (**stset survtime_var, failure(censoring_var)**. A Kaplan Meier graph by study arms will be presented with risk table, using the STATA command ^9^ **sts graph, by(armname) risktable(start_time(interval)end_time)**.

The relative risk between arms will be estimated by dividing the KM results at suitable timepoints, with 95%CI calculated using bootstrap method, non-attendance by the end of follow-up period is considered censored.

The results will be summarized in a table including the relative risk (RR) and 95% CI We conclude the following for:

- RR < 1, the attendance rate is decreased for the considered study arm compared to the reference category.
- RR = 1, no difference between the attendance rate of the considered study arm and the reference category.
- RR > 1, the attendance rate is increased for the considered study arm compared to the reference category.

## Subgroup analysis

The estimated attendance by 90 days and 180 days by arm will be reported by subgroups (with 95%CIs for each endpoint).

## Other analysis

Other exploratory analysis may be done to assess the potential risk of bias from administrative errors.

# Presentation of analysis

The results will be reported in the following tables and figures:

## Tables

[Table 1 Summary of characteristics of invitees by study arm.](#_Toc155961845)

[Table 2 **Summary of the characteristics of invitees invited by service.**](#_Toc155961846)

[Table 3 **Primary and secondary endpoints by study arms**](#_Toc155961847)

[Table 4 **Primary analysis of the endpoints**](#_Toc155961848)

[Table 5 **Secondary analysis of the endpoints**](#_Toc155961849)

[Table 6 Primary and secondary endpoint by subgroups.](#_Toc155961850)

## Figures

[Figure 1 Flow diagram of participants](#_Toc155961851)

[Figure 2 Kaplan Meier graph of the primary endpoint by study arms](#_Toc155961852)

[Figure 3 Kaplan Meier graph of the secondary endpoint by study arms](#_Toc155961853)

[Figure 4 Other Kaplan Meier graph of primary/secondary endpoints by subgroup of interest](#_Toc155961854)

# References

1. CONSORT. CONSORT 2010 checklist of information to include when reporting a randomised trial. Accessed 19^th^ December. Available from: <https://cdn-links.lww.com/permalink/phm/a/phm_00_00_2018_03_14_wu_ajpmr-d-17-00294_sdc1.pdf>
2. National statistics. English Indices of Deprivation 2019. Accessed 14^th^ December 2023. Available from: <https://www.gov.uk/government/statistics/english-indices-of-deprivation-2019>
3. Official Census and Labour Market Statistics. RM032 – Ethnic Group by sex by age. Accessed 4^th^ January 2024. Available from: <https://www.nomisweb.co.uk/query/construct/submit.asp?menuopt=201&subcomp=>
4. Office for National Statistics. Rural Urban Classification (2011) of Lower Layer Super Output Areas in England and Wales. Accessed 12^th^ December 2023. Available from: <https://geoportal.statistics.gov.uk/datasets/ons::rural-urban-classification-2011-of-lower-layer-super-output-areas-in-england-and-wales-1/explore>
5. Office for National Statistics. LSOA (2011) to LSOA (2021) to Local Authority District (2022) lookup for England and Wales. Accessed 12^th^ December 2023. Available from: <https://geoportal.statistics.gov.uk/datasets/ons::lsoa-2011-to-lsoa-2021-to-local-authority-district-2022-lookup-for-england-and-wales-version-2/explore>
6. StataCorp. Stata Statistical Software: Release 17.0. College Station, TX: StataCorp.
7. Gail MH, Tan WY, Piantadosi S. Tests for no treatment effect in randomized clinical trials. Biometrika 1988; 75: 57-64.
8. Wilson EB. Probable inference, the law of succession, and statistical inference. Journal of the American Statistical Association. 1927 Jun 1;22(158):209-12.
9. Lambert PC. Introduction to the stset command. 2006. University of Leicester. Accessed 19^th^ December 2023. Available from: <http://www.pauldickman.com/survival/stset.pdf>

# Appendices

See separate file for tables and figure.

## Data dictionary

| Variable | Definition |
| --- | --- |
| lsoacode | Layer Super Output Areas |
| uniqueid | Unique patient ID |
| mob | Month of birth |
| yob | Year of birth |
| ageatfoa | Age at first offered appointment in the screening episode |
| attendedappointment | Whether an appointment has been attended in this episode |
| closed | Whether the episode is closed yet |
| screeningdate | Date of the screening |
| reasonepisodeclosed | Reason for episode closure |
| Special requirement | Whether a screenee has special requirement(s) |
| previousscreeningdate | For incident screenees, the date of previous screening |
| armname | Arm of the study |
| batchid | Batch ID |
| prevalentincidentstatus | Prevalent or incidence screening episode |
| assessed |  |
| cancerregistrycandidate | Whether a screenee is also registered in cancer registry |
| letttype | Code for letter type sent |
| linkedletter | Code for previous letter sent |
| status | Letter printed/not |
| datecreated | Date the letter was created |
| printed_on | Date the letter was printed |
| clinic | Clinic code |
| date | Date of the first appointment offered/booked |
| time | Time of the screening appointment. |
| flag1999clinicnotanoi | Flag for administrative purpose |
| flag2oiinviteandnotaoicl | Flag for administrative purpose |
| flag3tabutanoiletterused | Flag for administrative purpose |
| warn | Warning flag for administrative purpose |
| oddorevenlastcharsx | Odd or even last character of SXnumber |
| oddorevenchar9nhs | Odd or even 9^th^ character of NHS number |
| tbc | Whether the site was hospital or mobile van |

Template for tables and figure

| Table 1 Summary of characteristics of invitees by study arm. | | | | | | | | | | | | | | | | | | | | | | | | | |
| --- | --- | --- | --- | --- | --- | --- | --- | --- | --- | --- | --- | --- | --- | --- | --- | --- | --- | --- | --- | --- | --- | --- | --- | --- | --- |
|  | | | | | Total | | | | TT | | | | | TO | | | | OT | | | | OO | | | |
|  | | | | | n | % | | | n | | % | | | n | | % | | n | % | | | n | | % | |
| Age in years, median (IQR) | | | | | XX.X | XX.X- XX.X | | | XX.X | | XX.X- XX.X | | | XX.X | | XX.X- XX.X | | XX.X | XX.X- XX.X | | | XX.X | | XX.X- XX.X | |
|  | | | | |  |  | | |  | |  | | |  | |  | |  |  | | |  | |  | |
| IMD quintiles | | | | |  |  | | |  | |  | | |  | |  | |  |  | | |  | |  | |
| 1: most deprived | | | | | XX | (XX.X%) | | | XX | | (XX.X%) | | | XX | | (XX.X%) | | XX | (XX.X%) | | | XX | | (XX.X%) | |
| 2 | | | | | XX | (XX.X%) | | | XX | | (XX.X%) | | | XX | | (XX.X%) | | XX | (XX.X%) | | | XX | | (XX.X%) | |
| 3 | | | | | XX | (XX.X%) | | | XX | | (XX.X%) | | | XX | | (XX.X%) | | XX | (XX.X%) | | | XX | | (XX.X%) | |
| 4 | | | | | XX | (XX.X%) | | | XX | | (XX.X%) | | | XX | | (XX.X%) | | XX | (XX.X%) | | | XX | | (XX.X%) | |
| 5: least deprived | | | | | XX | (XX.X%) | | | XX | | (XX.X%) | | | XX | | (XX.X%) | | XX | (XX.X%) | | | XX | | (XX.X%) | |
| Missing | | | | | XX | (XX.X%) | | | XX | | (XX.X%) | | | XX | | (XX.X%) | | XX | (XX.X%) | | | XX | | (XX.X%) | |
|  | | | | |  |  | | |  | |  | | |  | |  | |  |  | | |  | |  | |
| Ethnicity (area-level estimate) | | | | |  |  | | |  | |  | | |  | |  | |  |  | | |  | |  | |
| Asian or Asian British | | | | | XX | (XX.X%) | | | XX | | (XX.X%) | | | XX | | (XX.X%) | | XX | (XX.X%) | | | XX | | (XX.X%) | |
| Black or Black British | | | | | XX | (XX.X%) | | | XX | | (XX.X%) | | | XX | | (XX.X%) | | XX | (XX.X%) | | | XX | | (XX.X%) | |
| Mixed | | | | | XX | (XX.X%) | | | XX | | (XX.X%) | | | XX | | (XX.X%) | | XX | (XX.X%) | | | XX | | (XX.X%) | |
| Other ethnic groups | | | | | XX | (XX.X%) | | | XX | | (XX.X%) | | | XX | | (XX.X%) | | XX | (XX.X%) | | | XX | | (XX.X%) | |
| White | | | | | XX | (XX.X%) | | | XX | | (XX.X%) | | | XX | | (XX.X%) | | XX | (XX.X%) | | | XX | | (XX.X%) | |
| Missing | | | | | XX | (XX.X%) | | | XX | | (XX.X%) | | | XX | | (XX.X%) | | XX | (XX.X%) | | | XX | | (XX.X%) | |
|  | | | | |  |  | | |  | |  | | |  | |  | |  |  | | |  | |  | |
| Prevalence and Age | | | | |  |  | | |  | |  | | |  | |  | |  |  | | |  | |  | |
| Prevalent and under 60 | | | | | XX | (XX.X%) | | | XX | | (XX.X%) | | | XX | | (XX.X%) | | XX | (XX.X%) | | | XX | | (XX.X%) | |
| Incident and under 60 | | | | | XX | (XX.X%) | | | XX | | (XX.X%) | | | XX | | (XX.X%) | | XX | (XX.X%) | | | XX | | (XX.X%) | |
| 60 or over | | | | | XX | (XX.X%) | | | XX | | (XX.X%) | | | XX | | (XX.X%) | | XX | (XX.X%) | | | XX | | (XX.X%) | |
| Missing | | | | | XX | (XX.X%) | | | XX | | (XX.X%) | | | XX | | (XX.X%) | | XX | (XX.X%) | | | XX | | (XX.X%) | |
|  | | | | |  |  | | |  | |  | | |  | |  | |  |  | | |  | |  | |
| Service | | | | |  |  | | |  | |  | | |  | |  | |  |  | | |  | |  | |
| Central and East London (Pilot) | | | | | XX | (XX.X%) | | | XX | | (XX.X%) | | | XX | | (XX.X%) | | XX | (XX.X%) | | | XX | | (XX.X%) | |
| Outer NE London | | | | | XX | (XX.X%) | | | XX | | (XX.X%) | | | XX | | (XX.X%) | | XX | (XX.X%) | | | XX | | (XX.X%) | |
| North London | | | | | XX | (XX.X%) | | | XX | | (XX.X%) | | | XX | | (XX.X%) | | XX | (XX.X%) | | | XX | | (XX.X%) | |
| Cambridge | | | | | XX | (XX.X%) | | | XX | | (XX.X%) | | | XX | | (XX.X%) | | XX | (XX.X%) | | | XX | | (XX.X%) | |
| N&E Devon | | | | | XX | (XX.X%) | | | XX | | (XX.X%) | | | XX | | (XX.X%) | | XX | (XX.X%) | | | XX | | (XX.X%) | |
| Doncaster | | | | | XX | (XX.X%) | | | XX | | (XX.X%) | | | XX | | (XX.X%) | | XX | (XX.X%) | | | XX | | (XX.X%) | |
| Dudley, Wolverh and SW Staffords^b^ | | | | | XX | (XX.X%) | | | XX | | (XX.X%) | | | XX | | (XX.X%) | | XX | (XX.X%) | | | XX | | (XX.X%) | |
| Missing | | | | | XX | (XX.X%) | | | XX | | (XX.X%) | | | XX | | (XX.X%) | | XX | (XX.X%) | | | XX | | (XX.X%) | |
|  | | | | |  |  | | |  | |  | | |  | |  | |  |  | | |  | |  | |
| Site | | | | |  |  | | |  | |  | | |  | |  | |  |  | | |  | |  | |
| Static | | | | | XX | (XX.X%) | | | XX | | (XX.X%) | | | XX | | (XX.X%) | | XX | (XX.X%) | | | XX | | (XX.X%) | |
| Mobile | | | | | XX | (XX.X%) | | | XX | | (XX.X%) | | | XX | | (XX.X%) | | XX | (XX.X%) | | | XX | | (XX.X%) | |
| Missing | | | | | XX | (XX.X%) | | | XX | | (XX.X%) | | | XX | | (XX.X%) | | XX | (XX.X%) | | | XX | | (XX.X%) | |
|  | | | | |  |  | | |  | |  | | |  | |  | |  |  | | |  | |  | |
| Rurality | | | | | XX | (XX.X%) | | | XX | | (XX.X%) | | | XX | | (XX.X%) | | XX | (XX.X%) | | | XX | | (XX.X%) | |
| Rural | | | | | XX | (XX.X%) | | | XX | | (XX.X%) | | | XX | | (XX.X%) | | XX | (XX.X%) | | | XX | | (XX.X%) | |
| Urban | | | | | XX | (XX.X%) | | | XX | | (XX.X%) | | | XX | | (XX.X%) | | XX | (XX.X%) | | | XX | | (XX.X%) | |
| Missing | | | | |  |  | | |  | |  | | |  | |  | |  |  | | |  | |  | |
| a This is the episode outcome 3 years ago, only for second or subsequent episode.  b Dudley, Wolverhampton and Southwest Staffordshire. | | | | | | | | | | | | | | | | | | | | | | | | | |
| Table 2 **Summary of the characteristics of invitees invited by service.** | | | | | | | | | | | | | | | | | | | | | | | | | |
|  | Central and East London (Pilot) | | Outer NE London | | | | North London | | | Cambridge | | | N&E Devon | | | | Doncaster | | | Dudley, Wolverh and SW Staffords^b^ | | |  | | |
|  | n | % | n | % | | | n | % | | n | | % | n | | % | | n | % | | n | % | |  | |  |
| Age in years, median (IQR) | XX.X | XX.X- XX.X | XX.X | XX.X- XX.X | | | XX.X | XX.X- XX.X | | XX.X | | XX.X- XX.X | XX.X | | XX.X- XX.X | | XX.X | XX.X- XX.X | | XX.X | XX.X- XX.X | |  | |  |
|  |  |  |  |  | | |  |  | |  | |  |  | |  | |  |  | |  |  | |  | |  |
| IMD quintiles | XX | (XX.X%) | XX | (XX.X%) | | | XX | (XX.X%) | | XX | | (XX.X%) | XX | | (XX.X%) | | XX | (XX.X%) | | XX | (XX.X%) | |  | |  |
| 1: most deprived | XX | (XX.X%) | XX | (XX.X%) | | | XX | (XX.X%) | | XX | | (XX.X%) | XX | | (XX.X%) | | XX | (XX.X%) | | XX | (XX.X%) | |  | |  |
| 2 | XX | (XX.X%) | XX | (XX.X%) | | | XX | (XX.X%) | | XX | | (XX.X%) | XX | | (XX.X%) | | XX | (XX.X%) | | XX | (XX.X%) | |  | |  |
| 3 | XX | (XX.X%) | XX | (XX.X%) | | | XX | (XX.X%) | | XX | | (XX.X%) | XX | | (XX.X%) | | XX | (XX.X%) | | XX | (XX.X%) | |  | |  |
| 4 | XX | (XX.X%) | XX | (XX.X%) | | | XX | (XX.X%) | | XX | | (XX.X%) | XX | | (XX.X%) | | XX | (XX.X%) | | XX | (XX.X%) | |  | |  |
| 5: least deprived | XX | (XX.X%) | XX | (XX.X%) | | | XX | (XX.X%) | | XX | | (XX.X%) | XX | | (XX.X%) | | XX | (XX.X%) | | XX | (XX.X%) | |  | |  |
| Missing | XX | (XX.X%) | XX | (XX.X%) | | | XX | (XX.X%) | | XX | | (XX.X%) | XX | | (XX.X%) | | XX | (XX.X%) | | XX | (XX.X%) | |  | |  |
|  |  |  |  |  | | |  |  | |  | |  |  | |  | |  |  | |  |  | |  | |  |
| Ethnicity (area-level estimate) |  |  |  |  | | |  |  | |  | |  |  | |  | |  |  | |  |  | |  | |  |
| Asian or Asian British | XX | (XX.X%) | XX | (XX.X%) | | | XX | (XX.X%) | | XX | | (XX.X%) | XX | | (XX.X%) | | XX | (XX.X%) | | XX | (XX.X%) | |  | |  |
| Black or Black British | XX | (XX.X%) | XX | (XX.X%) | | | XX | (XX.X%) | | XX | | (XX.X%) | XX | | (XX.X%) | | XX | (XX.X%) | | XX | (XX.X%) | |  | |  |
| Mixed | XX | (XX.X%) | XX | (XX.X%) | | | XX | (XX.X%) | | XX | | (XX.X%) | XX | | (XX.X%) | | XX | (XX.X%) | | XX | (XX.X%) | |  | |  |
| Other ethnic groups | XX | (XX.X%) | XX | (XX.X%) | | | XX | (XX.X%) | | XX | | (XX.X%) | XX | | (XX.X%) | | XX | (XX.X%) | | XX | (XX.X%) | |  | |  |
| White | XX | (XX.X%) | XX | (XX.X%) | | | XX | (XX.X%) | | XX | | (XX.X%) | XX | | (XX.X%) | | XX | (XX.X%) | | XX | (XX.X%) | |  | |  |
| Missing | XX | (XX.X%) | XX | (XX.X%) | | | XX | (XX.X%) | | XX | | (XX.X%) | XX | | (XX.X%) | | XX | (XX.X%) | | XX | (XX.X%) | |  | |  |
|  |  |  |  |  | | |  |  | |  | |  |  | |  | |  |  | |  |  | |  | |  |
|  |  |  |  |  | | |  |  | |  | |  |  | |  | |  |  | |  |  | |  | |  |
|  |  |  |  |  | | |  |  | |  | |  |  | |  | |  |  | |  |  | |  | |  |
|  |  |  |  |  | | |  |  | |  | |  |  | |  | |  |  | |  |  | |  | |  |
|  |  |  |  |  | | |  |  | |  | |  |  | |  | |  |  | |  |  | |  | |  |
|  |  |  |  |  | | |  |  | |  | |  |  | |  | |  |  | |  |  | |  | |  |
| Prevalence and Age |  |  |  |  | | |  |  | |  | |  |  | |  | |  |  | |  |  | |  | |  |
| Prevalent and under 60 | XX | (XX.X%) | XX | (XX.X%) | | | XX | (XX.X%) | | XX | | (XX.X%) | XX | | (XX.X%) | | XX | (XX.X%) | | XX | (XX.X%) | |  | |  |
| Incident and under 60 | XX | (XX.X%) | XX | (XX.X%) | | | XX | (XX.X%) | | XX | | (XX.X%) | XX | | (XX.X%) | | XX | (XX.X%) | | XX | (XX.X%) | |  | |  |
| 60 or over | XX | (XX.X%) | XX | (XX.X%) | | | XX | (XX.X%) | | XX | | (XX.X%) | XX | | (XX.X%) | | XX | (XX.X%) | | XX | (XX.X%) | |  | |  |
| Missing | XX | (XX.X%) | XX | (XX.X%) | | | XX | (XX.X%) | | XX | | (XX.X%) | XX | | (XX.X%) | | XX | (XX.X%) | | XX | (XX.X%) | |  | |  |
|  |  |  |  |  | | |  |  | |  | |  |  | |  | |  |  | |  |  | |  | |  |
| Location |  |  |  |  | | |  |  | |  | |  |  | |  | |  |  | |  |  | |  | |  |
| Static | XX | (XX.X%) | XX | (XX.X%) | | | XX | (XX.X%) | | XX | | (XX.X%) | XX | | (XX.X%) | | XX | (XX.X%) | | XX | (XX.X%) | |  | |  |
| Mobile | XX | (XX.X%) | XX | (XX.X%) | | | XX | (XX.X%) | | XX | | (XX.X%) | XX | | (XX.X%) | | XX | (XX.X%) | | XX | (XX.X%) | |  | |  |
| Missing | XX | (XX.X%) | XX | (XX.X%) | | | XX | (XX.X%) | | XX | | (XX.X%) | XX | | (XX.X%) | | XX | (XX.X%) | | XX | (XX.X%) | |  | |  |
|  |  |  |  |  | | |  |  | |  | |  |  | |  | |  |  | |  |  | |  | |  |
| Rurality |  |  |  |  | | |  |  | |  | |  |  | |  | |  |  | |  |  | |  | |  |
| Rural | XX | (XX.X%) | XX | (XX.X%) | | | XX | (XX.X%) | | XX | | (XX.X%) | XX | | (XX.X%) | | XX | (XX.X%) | | XX | (XX.X%) | |  | |  |
| Urban | XX | (XX.X%) | XX | (XX.X%) | | | XX | (XX.X%) | | XX | | (XX.X%) | XX | | (XX.X%) | | XX | (XX.X%) | | XX | (XX.X%) | |  | |  |
| Missing | XX | (XX.X%) | XX | (XX.X%) | | | XX | (XX.X%) | | XX | | (XX.X%) | XX | | (XX.X%) | | XX | (XX.X%) | | XX | (XX.X%) | |  | |  |
|  |  |  |  |  | | |  |  | |  | |  |  | |  | |  |  | |  |  | |  | |  |
| a This is the episode outcome 3 years ago, only for second or subsequent episode.  b Dudley, Wolverhampton and Southwest Staffordshire. | | | | | | | | | | | | | | | | | | | | | | | | | |

| Table 3 **Primary and secondary endpoints by study arms** | | | | | | | |
| --- | --- | --- | --- | --- | --- | --- | --- |
| Study arm | Total invited | 90-day attendance | 90-day attendance (%) | 95%CI | 180-day attendance | 180-day attendance (%)* | 95%CI |
| TT | XX | XX | XX.X% | XX.X%, XX.X% | XX | XX.X% | XX.X%, XX.X% |
| OT | XX | XX | XX.X% | XX.X%, XX.X% | XX | XX.X% | XX.X%, XX.X% |
| TO | XX | XX | XX.X% | XX.X%, XX.X% | XX | XX.X% | XX.X%, XX.X% |
| OO | XX | XX | XX.X% | XX.X%, XX.X% | XX | XX.X% | XX.X%, XX.X% |
| Total | XX | XX | XX.X% | XX.X%, XX.X% | XX | XX.X% | XX.X%, XX.X% |
|  | | | | | | | |

*For the 180-day attendance, estimate from the KM graph, add the surv_table for this graph if possible.

| Table 4 **Primary analysis of the endpoints** | | | | | | |
| --- | --- | --- | --- | --- | --- | --- |
|  | Study arm | 90-day attendance | | | 180-day attendance* | |
|  |  | IRR | 95% CI | p-value ^a^ | RR | 95% CI |
| 1 | TT vs OO | X.XX | X.XX, X.XX | X.XXX | X.XX | X.XX, X.XX |
| 2 | TT vs OT | X.XX | X.XX, X.XX | X.XXX | X.XX | X.XX, X.XX |
| 3 | TO vs OT | X.XX | X.XX, X.XX | X.XXX | X.XX | X.XX, X.XX |
| 4 | TT VS TO | X.XX | X.XX, X.XX | X.XXX | X.XX | X.XX, X.XX |
| 5 | OT vs OO | X.XX | X.XX, X.XX | X.XXX | X.XX | X.XX, X.XX |
| IRR: incidence-rate ratio, CI: confidence interval, RR: relative risk. a. Report p-value to 2sf.  *For the 180-day attendance, estimate from the KM graph. | | | | | | |

| Table 5 **Secondary analysis of the endpoints** | | | | | |
| --- | --- | --- | --- | --- | --- |
|  | 90-day attendance | | | 180-day attendance* | |
|  | IRR | 95% CI | p-value ^a^ | RR | 95% CI |
| (TT+TO) vs (OT+OO) | X.XX | X.XX, X.XX | X.XXX | X.XX | X.XX, X.XX |
| (TT+OT) vs (TO+OO) | X.XX | X.XX, X.XX | X.XXX | X.XX | X.XX, X.XX |
| IRR: incidence-rate ratio, CI: confidence interval, RR: relative risk. a. Report p-value to 2sf.  *For the 180-day attendance, estimate from the KM graph. | | | | | |

| Table 6 Primary and secondary endpoint by subgroups. | | | | | |  |  |  |
| --- | --- | --- | --- | --- | --- | --- | --- | --- |
| Subgroup | Study arm | Total invited | 90-day attendance | 90-day attendance (%) | 95%CI | 180-day attendance | 180-day attendance (%) | 95%CI |
| IMD quintile |  |  |  |  |  |  |  |  |
| 1 | TT | XX | XX | XX.X% | XX.X%, XX.X% | XX | XX.X% | XX.X%, XX.X% |
|  | OT | XX | XX | XX.X% | XX.X%, XX.X% | XX | XX.X% | XX.X%, XX.X% |
|  | TO | XX | XX | XX.X% | XX.X%, XX.X% | XX | XX.X% | XX.X%, XX.X% |
|  | OO | XX | XX | XX.X% | XX.X%, XX.X% | XX | XX.X% | XX.X%, XX.X% |
|  |  |  |  |  |  |  |  |  |
| 2 | TT | XX | XX | XX.X% | XX.X%, XX.X% | XX | XX.X% | XX.X%, XX.X% |
|  | OT | XX | XX | XX.X% | XX.X%, XX.X% | XX | XX.X% | XX.X%, XX.X% |
|  | TO | XX | XX | XX.X% | XX.X%, XX.X% | XX | XX.X% | XX.X%, XX.X% |
|  | OO | XX | XX | XX.X% | XX.X%, XX.X% | XX | XX.X% | XX.X%, XX.X% |
|  |  |  |  |  |  |  |  |  |
| 3 | TT | XX | XX | XX.X% | XX.X%, XX.X% | XX | XX.X% | XX.X%, XX.X% |
|  | OT | XX | XX | XX.X% | XX.X%, XX.X% | XX | XX.X% | XX.X%, XX.X% |
|  | TO | XX | XX | XX.X% | XX.X%, XX.X% | XX | XX.X% | XX.X%, XX.X% |
|  | OO | XX | XX | XX.X% | XX.X%, XX.X% | XX | XX.X% | XX.X%, XX.X% |
|  |  |  |  |  |  |  |  |  |
| 4 | TT | XX | XX | XX.X% | XX.X%, XX.X% | XX | XX.X% | XX.X%, XX.X% |
|  | OT | XX | XX | XX.X% | XX.X%, XX.X% | XX | XX.X% | XX.X%, XX.X% |
|  | TO | XX | XX | XX.X% | XX.X%, XX.X% | XX | XX.X% | XX.X%, XX.X% |
|  | OO | XX | XX | XX.X% | XX.X%, XX.X% | XX | XX.X% | XX.X%, XX.X% |
|  |  |  |  |  |  |  |  |  |
| 5 | TT | XX | XX | XX.X% | XX.X%, XX.X% | XX | XX.X% | XX.X%, XX.X% |
|  | OT | XX | XX | XX.X% | XX.X%, XX.X% | XX | XX.X% | XX.X%, XX.X% |
|  | TO | XX | XX | XX.X% | XX.X%, XX.X% | XX | XX.X% | XX.X%, XX.X% |
|  | OO | XX | XX | XX.X% | XX.X%, XX.X% | XX | XX.X% | XX.X%, XX.X% |
|  |  |  |  |  |  |  |  |  |
| Ethnicity  (area level estimate) |  |  |  |  |  |  |  |  |
| White | TT | XX | XX | XX.X% | XX.X%, XX.X% | XX | XX.X% | XX.X%, XX.X% |
|  | OT | XX | XX | XX.X% | XX.X%, XX.X% | XX | XX.X% | XX.X%, XX.X% |
|  | TO | XX | XX | XX.X% | XX.X%, XX.X% | XX | XX.X% | XX.X%, XX.X% |
|  | OO | XX | XX | XX.X% | XX.X%, XX.X% | XX | XX.X% | XX.X%, XX.X% |
|  |  |  |  |  |  |  |  |  |
| Non-white | TT | XX | XX | XX.X% | XX.X%, XX.X% | XX | XX.X% | XX.X%, XX.X% |
|  | OT | XX | XX | XX.X% | XX.X%, XX.X% | XX | XX.X% | XX.X%, XX.X% |
|  | TO | XX | XX | XX.X% | XX.X%, XX.X% | XX | XX.X% | XX.X%, XX.X% |
|  | OO | XX | XX | XX.X% | XX.X%, XX.X% | XX | XX.X% | XX.X%, XX.X% |
|  |  |  |  |  |  |  |  |  |
| Prevalence and Age |  |  |  |  |  |  |  |  |
| Prevalent and under 60 | TT | XX | XX | XX.X% | XX.X%, XX.X% | XX | XX.X% | XX.X%, XX.X% |
|  | OT | XX | XX | XX.X% | XX.X%, XX.X% | XX | XX.X% | XX.X%, XX.X% |
|  | TO | XX | XX | XX.X% | XX.X%, XX.X% | XX | XX.X% | XX.X%, XX.X% |
|  | OO | XX | XX | XX.X% | XX.X%, XX.X% | XX | XX.X% | XX.X%, XX.X% |
|  |  |  |  |  |  |  |  |  |
| Incident and under 60 | TT | XX | XX | XX.X% | XX.X%, XX.X% | XX | XX.X% | XX.X%, XX.X% |
|  | OT | XX | XX | XX.X% | XX.X%, XX.X% | XX | XX.X% | XX.X%, XX.X% |
|  | TO | XX | XX | XX.X% | XX.X%, XX.X% | XX | XX.X% | XX.X%, XX.X% |
|  | OO | XX | XX | XX.X% | XX.X%, XX.X% | XX | XX.X% | XX.X%, XX.X% |
|  |  |  |  |  |  |  |  |  |
| 60 or over | TT | XX | XX | XX.X% | XX.X%, XX.X% | XX | XX.X% | XX.X%, XX.X% |
|  | OT | XX | XX | XX.X% | XX.X%, XX.X% | XX | XX.X% | XX.X%, XX.X% |
|  | TO | XX | XX | XX.X% | XX.X%, XX.X% | XX | XX.X% | XX.X%, XX.X% |
|  | OO | XX | XX | XX.X% | XX.X%, XX.X% | XX | XX.X% | XX.X%, XX.X% |
|  |  |  |  |  |  |  |  |  |
| Service |  |  |  |  |  |  |  |  |
| Central and East London (Pilot) | TT | XX | XX | XX.X% | XX.X%, XX.X% | XX | XX.X% | XX.X%, XX.X% |
|  | OT | XX | XX | XX.X% | XX.X%, XX.X% | XX | XX.X% | XX.X%, XX.X% |
|  | TO | XX | XX | XX.X% | XX.X%, XX.X% | XX | XX.X% | XX.X%, XX.X% |
|  | OO | XX | XX | XX.X% | XX.X%, XX.X% | XX | XX.X% | XX.X%, XX.X% |
|  |  |  |  |  |  |  |  |  |
| Outer NE London | TT | XX | XX | XX.X% | XX.X%, XX.X% | XX | XX.X% | XX.X%, XX.X% |
|  | OT | XX | XX | XX.X% | XX.X%, XX.X% | XX | XX.X% | XX.X%, XX.X% |
|  | TO | XX | XX | XX.X% | XX.X%, XX.X% | XX | XX.X% | XX.X%, XX.X% |
|  | OO | XX | XX | XX.X% | XX.X%, XX.X% | XX | XX.X% | XX.X%, XX.X% |
|  |  |  |  |  |  |  |  |  |
| North London | TT | XX | XX | XX.X% | XX.X%, XX.X% | XX | XX.X% | XX.X%, XX.X% |
|  | OT | XX | XX | XX.X% | XX.X%, XX.X% | XX | XX.X% | XX.X%, XX.X% |
|  | TO | XX | XX | XX.X% | XX.X%, XX.X% | XX | XX.X% | XX.X%, XX.X% |
|  | OO | XX | XX | XX.X% | XX.X%, XX.X% | XX | XX.X% | XX.X%, XX.X% |
|  |  |  |  |  |  |  |  |  |
| Cambridge | TT | XX | XX | XX.X% | XX.X%, XX.X% | XX | XX.X% | XX.X%, XX.X% |
|  | OT | XX | XX | XX.X% | XX.X%, XX.X% | XX | XX.X% | XX.X%, XX.X% |
|  | TO | XX | XX | XX.X% | XX.X%, XX.X% | XX | XX.X% | XX.X%, XX.X% |
|  | OO | XX | XX | XX.X% | XX.X%, XX.X% | XX | XX.X% | XX.X%, XX.X% |
|  |  |  |  |  |  |  |  |  |
| N&E Devon | TT | XX | XX | XX.X% | XX.X%, XX.X% | XX | XX.X% | XX.X%, XX.X% |
|  | OT | XX | XX | XX.X% | XX.X%, XX.X% | XX | XX.X% | XX.X%, XX.X% |
|  | TO | XX | XX | XX.X% | XX.X%, XX.X% | XX | XX.X% | XX.X%, XX.X% |
|  | OO | XX | XX | XX.X% | XX.X%, XX.X% | XX | XX.X% | XX.X%, XX.X% |
|  |  |  |  |  |  |  |  |  |
| Doncaster | TT | XX | XX | XX.X% | XX.X%, XX.X% | XX | XX.X% | XX.X%, XX.X% |
|  | OT | XX | XX | XX.X% | XX.X%, XX.X% | XX | XX.X% | XX.X%, XX.X% |
|  | TO | XX | XX | XX.X% | XX.X%, XX.X% | XX | XX.X% | XX.X%, XX.X% |
|  | OO | XX | XX | XX.X% | XX.X%, XX.X% | XX | XX.X% | XX.X%, XX.X% |
|  |  |  |  |  |  |  |  |  |
| Dudley, Wolverhampton and SW Staffordshire | TT | XX | XX | XX.X% | XX.X%, XX.X% | XX | XX.X% | XX.X%, XX.X% |
|  | OT | XX | XX | XX.X% | XX.X%, XX.X% | XX | XX.X% | XX.X%, XX.X% |
|  | TO | XX | XX | XX.X% | XX.X%, XX.X% | XX | XX.X% | XX.X%, XX.X% |
|  | OO | XX | XX | XX.X% | XX.X%, XX.X% | XX | XX.X% | XX.X%, XX.X% |
| Location |  |  |  |  |  |  |  |  |
| Static | TT | XX | XX | XX.X% | XX.X%, XX.X% | XX | XX.X% | XX.X%, XX.X% |
|  | OT | XX | XX | XX.X% | XX.X%, XX.X% | XX | XX.X% | XX.X%, XX.X% |
|  | TO | XX | XX | XX.X% | XX.X%, XX.X% | XX | XX.X% | XX.X%, XX.X% |
|  | OO | XX | XX | XX.X% | XX.X%, XX.X% | XX | XX.X% | XX.X%, XX.X% |
|  |  |  |  |  |  |  |  |  |
| Mobile | TT | XX | XX | XX.X% | XX.X%, XX.X% | XX | XX.X% | XX.X%, XX.X% |
|  | OT | XX | XX | XX.X% | XX.X%, XX.X% | XX | XX.X% | XX.X%, XX.X% |
|  | TO | XX | XX | XX.X% | XX.X%, XX.X% | XX | XX.X% | XX.X%, XX.X% |
|  | OO | XX | XX | XX.X% | XX.X%, XX.X% | XX | XX.X% | XX.X%, XX.X% |
|  |  |  |  |  |  |  |  |  |
| Rurality |  |  |  |  |  |  |  |  |
| Rural | TT | XX | XX | XX.X% | XX.X%, XX.X% | XX | XX.X% | XX.X%, XX.X% |
|  | OT | XX | XX | XX.X% | XX.X%, XX.X% | XX | XX.X% | XX.X%, XX.X% |
|  | TO | XX | XX | XX.X% | XX.X%, XX.X% | XX | XX.X% | XX.X%, XX.X% |
|  | OO | XX | XX | XX.X% | XX.X%, XX.X% | XX | XX.X% | XX.X%, XX.X% |
|  |  |  |  |  |  |  |  |  |
| Rural | TT | XX | XX | XX.X% | XX.X%, XX.X% | XX | XX.X% | XX.X%, XX.X% |
|  | OT | XX | XX | XX.X% | XX.X%, XX.X% | XX | XX.X% | XX.X%, XX.X% |
|  | TO | XX | XX | XX.X% | XX.X%, XX.X% | XX | XX.X% | XX.X%, XX.X% |
|  | OO | XX | XX | XX.X% | XX.X%, XX.X% | XX | XX.X% | XX.X%, XX.X% |
|  | | | | | |  |  |  |

Figure 1 Flow diagram of participants

Allocation (second letter)

Pseudorandomised (n=x,xxx)

**Open invitation**

- special requirement (n=xx)

- other exclusion criteria (n=xx)

**Timed appointment**

- special requirement (n=xx)

- other exclusion criteria (n=xx)

**Timed appointment**

- special requirement (n=xx)

- other exclusion criteria (n=xx)

**Open invitation**

- special requirement (n=xx)

- other exclusion criteria (n=xx)

Exclusion (n=xx)

- Lost to follow-up, e.g. women moved address (n=xx)

- Opt out screening

- Other reasons

Exclusion (n=xx)

- Lost to follow-up, e.g. women moved address (n=xx)

- Opt out screening

- Other reasons

Exclusion (n=xx)

- Lost to follow-up, e.g. women moved address (n=xx)

- Opt out screening

- Other reasons

Analysis

Exclusion (n=xx)

- special requirement (n=xx)

- other exclusion criteria (n=xx)

- wrong letters sent (n=xx)

- other admin errors (n=xx)

- other reasons (n=xx)

Exclusion (n=xx)

- special requirement (n=xx)

- other exclusion criteria (n=xx)

- wrong letters sent (n=xx)

- other admin errors (n=xx)

- other reasons (n=xx)

Exclusion (n=xx)

- special requirement (n=xx)

- other exclusion criteria (n=xx)

- wrong letters sent (n=xx)

- other admin errors (n=xx)

- other reasons (n=xx)

Exclusion (n=xx)

- special requirement (n=xx)

- other exclusion criteria (n=xx)

- wrong letters sent (n=xx)

- other admin errors (n=xx)

- other reasons (n=xx)

Follow-up

Exclusion (n=xx)

- Lost to follow-up, e.g. women moved address (n=xx)

- Opt out screening

- Other reasons

Open invitation

- special requirement (n=xx)
- other exclusion criteria (n=xx)

Timed appointment

- special requirement (n=xx)
- other exclusion criteria (n=xx)

Allocation (first letter)

Figure 2 Kaplan Meier graph of the primary endpoint by study arms

Figure 3 Kaplan Meier graph of the secondary endpoint by study arms

Figure 4 Other Kaplan Meier graph of primary/secondary endpoints by subgroup of interest

**References**

1. Office for National Statistics. Population Estimates for UK, England and Wales, Scotland and Northern Ireland: Mid-2019-april-2020-geography. . 2024.

2. Office for National Statistics. Ethnic group classifications: Census 2021.

3. NHS Digital. NHS Breast Screening Programme, England 2021-22. 2023. <https://digital.nhs.uk/data-and-information/publications/statistical/breast-screening-programme/england---2021-22/mainreport2122-copy#section-4-uptake-of-invitations> (accessed 11/11/2024.
